# Corporate power and the international trade regime preventing progressive policy action on non-communicable diseases: a realist review

**DOI:** 10.1093/heapol/czaa148

**Published:** 2020-12-04

**Authors:** Penelope Milsom, Richard Smith, Phillip Baker, Helen Walls

**Affiliations:** Department of Global Health and Development, Faculty of Public Health and Policy, London School of Hygiene and Tropical Medicine, 15-17 Tavistock Place, Kings Cross, London WC1H 9SH, UK; College of Medicine and Health, University of Exeter, Magdalen Road, Exeter, EX1 2LU, UK; Institute for Physical Activity and Nutrition, School of Exercise and Nutrition Sciences, Deakin University, 221 Burwood Highway, Burwood, Melbourne, Victoria 3125 Australia; Department of Global Health and Development, Faculty of Public Health and Policy, London School of Hygiene and Tropical Medicine, 15-17 Tavistock Place, Kings Cross, London WC1H 9SH, UK

**Keywords:** Trade agreements, trade liberalization, public health policy, political economy, power analysis

## Abstract

Transnational tobacco, alcohol and ultra-processed food corporations use the international trade regime to prevent policy action on non-communicable diseases (NCDs); i.e. to promote policy ‘non-decisions’. Understanding policy non-decisions can be assisted by identifying power operating in relevant decision-making spaces, but trade and health research rarely explicitly engages with theories of power. This realist review aimed to synthesize evidence of different forms and mechanisms of power active in trade and health decision-making spaces to understand better why NCD policy non-decisions persist and the implications for future transformative action. We iteratively developed power-based theories explaining how transnational health-harmful commodity corporations (THCCs) utilize the international trade regime to encourage NCD policy non-decisions. To support theory development, we also developed a conceptual framework for analysing power in public health policymaking. We searched six databases and relevant grey literature and extracted, synthesized and mapped the evidence against the proposed theories. One hundred and four studies were included. Findings were presented for three key forms of power. Evidence indicates THCCs attempt to exercise instrumental power by extensive lobbying often via privileged access to trade and health decision-making spaces. When their legitimacy declines, THCCs have attempted to shift decision-making to more favourable international trade legal venues. THCCs benefit from structural power through the institutionalization of their involvement in health and trade agenda-setting processes. In terms of discursive power, THCCs effectively frame trade and health issues in ways that echo and amplify dominant neoliberal ideas. These processes may further entrench the individualization of NCDs, restrict conceivable policy solutions and perpetuate policymaking norms that privilege economic/trade interests over health. This review identifies different forms and mechanisms of power active in trade and health policy spaces that enable THCCs to prevent progressive action on NCDs. It also points to potential strategies for challenging these power dynamics and relations.

KEY MESSAGES

- The international trade regime provides transnational health-harmful commodity corporations with opportunities to use and benefit from instrumental, structural and discursive power in ways that likely contribute to non-communicable disease (NCD) prevention policy non-decisions in both national and international policymaking spaces—and particularly so under a dominant neoliberal paradigm.
- Recognizing power in all its forms across different political spaces and levels is essential for enabling public health actors to identify and evaluate effective strategies for improving trade and health policy coherence.

## Introduction

Understanding how corporations constrain public health policy action, or in other words, promote policy ‘non-decision-making’, has been a growing concern for nearly half a century. Over the past few decades, public health researchers have exposed multiple strategies used by transnational health-harmful commodity corporations (THCCs) to prevent regulation of health-harmful commodities (Jiang and Ling, 2013; Ulucanlar *et al.*, 2014; Gilmore *et al.*, 2015; Balwicki *et al.*, 2016; Bertscher *et al.*, 2018; Mccambridge *et al.*, 2018; Hawkins *et al.*, 2019). One increasingly relevant tactical area relates to international trade. Scholars have focused on analysing corporate use of trade rules and disputes, finding that by shaping trade rules, THCCs can limit future domestic public health policy space for regulating health-harmful commodities (Labonte *et al.*, 2011; Baker *et al.*, 2014) and by threatening or triggering a trade dispute it may be possible to generate regulatory chill across multiple countries (Hawkins *et al.*, 2019). These analyses have led to calls by public health advocates for transparency and accountability in trade agreement processes with greater participation of health actors; and ensured protection of public health policy space in trade agreements (McNeill *et al.*, 2017b). But little in practice has been achieved to transform patterns of exclusion of public health actors and concerns in trade policy development (Townsend *et al.*, 2020a). We argue this may be in part due to a failure to expose and adopt strategies that challenge the underlying power dynamics and relations at the nexus of trade and health.

Understanding the nature and mechanisms of power is increasingly recognized as critical to understanding contemporary public health policy processes and outcomes (Hansen *et al.*, 2013; Shiffman, 2014; Forman, 2015; Gómez, 2016; Gore and Parker, 2019), including non-decisions. Yet trade and health policy analysis has rarely engaged directly with theories of power. Only limited more recent empirical research has adopted a politically informed approach that examines certain aspects of power operating at the nexus of trade and health policy (Battams and Townsend, 2018; Lencucha *et al.*, 2018; Thow *et al.*, 2018). Research on framing in trade policy has described how a dominant neoliberal discourse privileges export interests over health (Townsend *et al.*, 2020b), including transnational ultra-processed food and alcohol exporters (Baker *et al.*, 2019). Studies have also explored strategies used by public health advocates to claim authority and legitimacy in trade negotiations (Townsend *et al.*, 2019). Other analyses have highlighted power asymmetries in access to decision-making spaces between business and public health actors (Battams and Townsend, 2018).

We suggest a more explicit and rigorous integration of theories of power in trade and health policy analyses could expand our understanding of how and why non-communicable disease (NCD) policy non-decisions persist as well as why, so far, relatively limited progress has been made towards increasing attention to NCD risk factors in trade policy. By making visible the different forms, mechanisms and spaces of power at the nexus of trade and health, it becomes possible to identify and evaluate strategies that may generate the necessary changes in power relations between health, trade and corporate actors to drive transformative policy change (Gaventa, 2006).

This realist review attempts to fill this gap in the literature. Building on established theories of power, we develop a conceptual framework for analysing the interrelationship between different forms, mechanisms and spaces of power in health policymaking. We then map existing evidence against theories grounded in the framework with the aim of better understanding how the power relations between trade, health and corporate actors have emerged and as such, why NCD policy non-decisions persist. By exposing power in this way, it also becomes possible to start identifying strategies to effectively challenge it. While evidence is included from countries across all income groups, we focus, where possible, on low- and middle-income countries (LMICs) since they have become the focus for expansion by many THCCs ( Lawrence, 2011; Savell *et al.*, 2015; Walls *et al.*, 2020) but generally have limited capacity—financial, institutional, technical and strategic—to resist attempts by THCCs’ to influence health policy processes (Walls *et al.*, 2015).

## Methods

The realist review methodology is based on identifying, interpreting and synthesizing a wide range of evidence to develop and refine explanatory theories about how and why a complex situation results in specific outcomes in certain contexts (Punton *et al.*, 2016). Thus, it is useful for expanding trade and health policy analysis beyond a description of problematic trade rules, towards gaining insights into the political economy of trade and health policy.

The review was undertaken according to an adapted protocol based broadly on Pawson’s five iterative stages: identifying and articulating the explanatory theories; searching for and appraising the evidence; extracting the data; synthesizing the evidence; and drawing conclusions (Pawson *et al.*, 2004). However, during stage one, we integrated an additional step of conceptual framework development. Here, based on synthesis of existing substantive theory relating to health policy processes, we developed a conceptual framework for analysing health policy decisions and non-decisions. The substantive theories embedded within the framework were used to facilitate explanatory theory development and ensure theory robustness. The reporting of this review adheres to RAMSES publication standards (Wong et al., 2013).

### Initial scope of the literature and explanatory theory development

Initial explanatory theories were developed through a rapid scoping of relevant trade and health policy literature. This was conducted using concept searches, e.g. ‘regulatory/policy chill’, ‘policy space’ or ‘trade and health policymaking’ in Scopus and Google Scholar, citation tracking and snowballing. Grey literature was also searched, and key studies suggested by other trade and health researchers known to the authors were sourced. Relevant explanatory information from different sources was interpreted, synthesized and mapped against the conceptual framework in an iterative process of preliminary theory development.

### Development of conceptual framework for analysing power in public health policymaking

**Figure 1:**
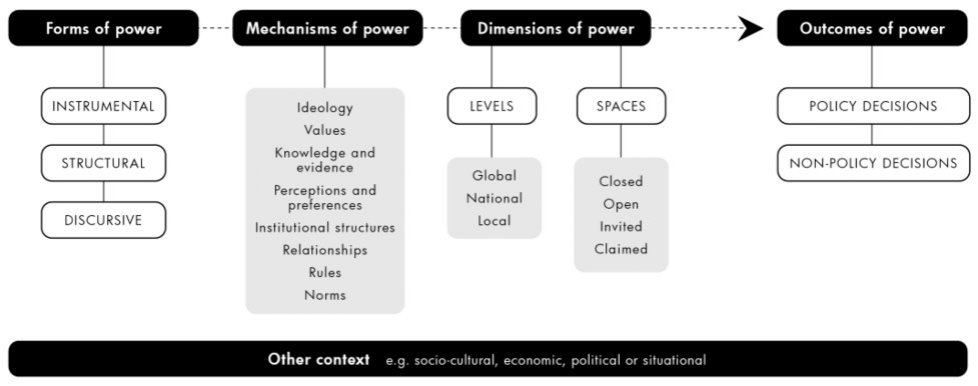
Conceptual framework for analysing power in public health policymaking.

Existing conceptual frameworks and theories useful for understanding the underlying causal mechanisms of contemporary health policy processes that were judged to be grounded, at least to some extent, in political economy theory, or included concepts of power, were identified through purposive searching (Bachrach and Baratz, 1962; Easton, 1965; Lukes, 1974; Sabatier and Jenkins-Smith, 1993; Walt and Gilson, 1994; John, 1998; Gaventa, 2006; Fuchs and Lederer, 2007; Shiffman and Smith, 2007; Howlett *et al.*, 2009; Rushton and Williams, 2012; Madureira Lima and Galea, 2018). In a process running parallel to explanatory theory building, we synthesized relevant elements from several of these frameworks and theories in an iterative process to develop a conceptual framework for analysing power in contemporary public health policymaking (Figure 1). The new conceptual framework builds on the three key forms of power outlined in Fuchs and Lederer’s framework with a strong focus on Lukes’ Three Dimensions of Power (Lukes, 1974). Each form of power is expressed via various mechanisms adapted from the ‘Three Is’ framework (Hall, 1997; Lavis *et al.*, 2002; Gauvin, 2014; Shearer *et al.*, 2016) and with examples drawn from Madureira Lima and Galea’s framework of corporate practices and health. Mechanisms are active in different spaces and at different levels as described in Gaventa’s Power Cube (Gaventa, 2006). Outcomes of power can be either policy decisions to act or non-decisions expressed as inaction. Specifically, the new conceptual framework was designed for analysing why and how certain public health issues and solutions are recognized and lead to meaningful policy action while others are either never recognized, suffocated before they make it onto the political agenda or are minimized or re-interpreted in the decision-making stage such that transformative policy action rarely occurs. The purpose of this was to further develop relevant substantive theory in which our explanatory theories could be grounded. We then mapped existing evidence found in the formal literature search against these theories derived from the framework.

Although the *forms*, *mechanisms*, *dimensions* and *outcomes* of power are diagrammatically presented in Figure 1 as separate elements, there is interdependence with dynamic feedback both within and between elements. Furthermore, multiple forms of power usually influence any given policy process.

Instrumental power is similar to Lukes’ first dimension of power and is focused on the direct influence different actors have over formal political decisions. Actor A is considered to have power over actor B if actor A can persuade actor B to do something she/he would not otherwise do (Lukes, 1974). For example, corporations use political financing to build relationships with politicians and undertake extensive lobbying to directly influence political decision-makers.

Structural power is generally hidden and includes setting the political agenda (Lukes, 1974; Gaventa, 2006). This is achieved by powerful actors reinforcing and taking advantage of social and political values, economic structures and institutional practices that limit the issues for consideration, who is included in decision-making spaces, and the scope of potential solutions (Bachrach and Baratz, 1962; Fuchs and Lederer, 2007). As a consequence, certain actors are prevented from raising to the political agenda issues that may be detrimental to more powerful actors who seek to defend the status quo (Fuchs and Lederer, 2007). For example, Tobacco control does not make it onto the political agenda in certain tobacco-producing countries. The second aspect of structural power refers to rule-setting power whereby underlying economic and institutional structures and processes place certain actors in the position of being able to make rules themselves (Fuchs and Lederer, 2007). For example, public–private partnerships enable corporations to influence the design, implementation and enforcement of certain rules, including via self-regulation schemes (Fuchs and Lederer, 2007).

Discursive power is the most insidious form of power and shapes the ideational and psychological boundaries of participation with significant problems and potential solutions not only kept from the decision-making table, but also outside the minds of actors involved, including those directly affected by the problem (Gaventa, 2006). Controlling how individuals perceive the world, shape their interpretation and understanding of important issues and preferred solutions (Lukes, 1974; Fuchs and Lederer, 2007). As such, less powerful actors are prevented from elevating significant policy issues and/or potential solutions in their own real interest because they are inconceivable, considered unacceptable or because they accept the status quo as natural and unchangeable or are socialized into believing an alternative is more beneficial (Lukes, 1974; Gaventa, 2006).

Groups of individual actors perceived as legitimate may strategically exercise discursive power (Fuchs and Lederer, 2007), e.g. the alcohol industry widely communicates an individual-level framing and narratives of alcohol-related harm, effectively excluding supply-side solutions as conceivable options. However, discursive power also emerges at the system level as a function of dominant ideas and institutional arrangements/practices that over time generate powerful cognitive and behavioural norms.

Each form of power may be exercised by actors or emerge from the system via eight different but interdependent mechanisms. These are ideologies (e.g. the neoliberal political ‘project’); values (e.g. individual freedom and choice); knowledge and evidence (e.g. ‘science to specification’, funding education and manufacturing doubt); perception and preference-shaping (e.g. issue framing and narratives communicated through corporate foundations, front groups, think tanks and public relations companies, opinion leaders, media capture and marketing and advertising); organizational structures (e.g. corporate participation in government agencies, committees and commissions and in policy development); relationships (e.g. corporate lobbying, revolving doors and political donations); rules (e.g. trade agreements and investment treaties); and norms (e.g. prioritization of economic over health imperatives in political decision-making).

*Dimensions* of power include the different *levels*—international, national or sub-national where power resides or is contested. Dimensions of power also include different *spaces*, defined here as formal or informal opportunities where actors can ‘potentially affect policies, discourses, decisions and relationships’ relevant to their interests (Gaventa, 2006). Spaces may be closed, open, invited or claimed and are interdependent, changing over time as actors and ideas struggle for legitimacy (Gaventa, 2006). The drivers of ill-health are increasingly recognized to arise from supra-national policy decisions beyond the control of national governments (Ottersen *et al.*, 2014). At the same time, power over such decisions can reside in spaces closed to health actors, both formal spaces e.g. the World Trade Organization (WTO) forums, and informal spaces, e.g. private meetings between industry and government.

The *outcome* of power may be a *policy decision* defined here simply as policy action. This may be voluntarily or involuntary and optimal or suboptimal, e.g. adopting a 10% tax on sugar-sweetened beverages rather than a preferred 20% tax evidenced to have a more optimal impact on consumption. The alternative outcome is a policy *non-decision* which is defined in this work as a voluntary decision not to act (e.g. deliberate prioritization of economic over health objectives); an involuntary failure to act (e.g. health actors do not pursue a desired measure to avoid a trade dispute); or inaction due to a psychological boundary issue (e.g. supply-side issues are never considered by policy actors since they so strongly contravene dominant perceptions of NCDs as an individual risk and responsibility issue).

Finally, certain *contexts*—political, economic, socio-cultural or situational—can inhibit or activate different mechanisms of power generating different outcomes. For example, LMICs very often have limited capacities—human, financial, organizational, technical and strategic—to exercise instrumental power in relation to negotiating trade rules or agreements in such a way that balances both their economic and health objectives. Lobbying as a form of instrumental power may be constrained where there are clear processes for managing conflicts of interest or restrictions on lobbying in governance spaces. The rule-setting (structural) power of THCCs may be enabled in contexts where there is a strong preference for market-led approaches to governance. Discourses that promote the primacy of markets and involvement of private sector in governance may be resisted in country contexts with strong human rights norms.

### Searching and appraising the evidence

#### Main search

A systematic search of the literature was undertaken with the aim of identifying the most relevant evidence to support or dispute the initial set of explanatory theories. The final search strategy included combinations of search and indexed terms for the concepts of international trade and investment liberalization, regulatory chill, policy process, relevant transnational corporations and three trade-sensitive public health policy areas: nutrition, tobacco control and alcohol regulation (Supplementary Text SI). These concepts were developed and refined iteratively with repeated testing in MEDLINE, review of search results, development/refinement of explanatory theories and, in turn, further concept development. The search terms were then developed through repeated testing in six databases: MEDLINE, Global Health, Econlit, SCOPUS, Web of Science and PubMed in order to balance reasonable sensitivity and specificity (given project time constraints) and the realist approach of searching broadly.

All six database searches were conducted in January 2020 and limited to English language publications between 1 January 2008 and 15 January 2020. It was considered reasonable to limit the search from 2008 onwards given that engagement with and understanding of trade issues by health academics was relatively limited prior to this (Smith *et al.*, 2009). Bibliography searching was conducted on studies particularly relevant for theory development. The final reference list was reviewed to ensure all relevant papers identified in the initial scoping review were included.

We also conducted searches for relevant grey literature in Google and Google Scholar and online repositories of the World Health Organization (WHO), WTO, United Nations Conference on Trade and Development and International Institute of Sustainable Development. All articles were downloaded to an *Endnote* database and duplicates removed.

#### Inclusion criteria

Pawson (2006) suggests that inclusion be based on relevance to programme theories and explanatory potential, whether the source material contains discernible ‘nuggets’ of evidence, and evidence of trustworthiness, or, in other words, ‘whether it is good and relevant enough’. Consistent with Pawson’s approach, no study was excluded based on a single aspect of quality. The criteria applied are outlined in Table 1.

**Table 1:** Inclusion criteria

Include the study if: It contains ‘nuggets’ of evidence that provide insight into the review questions, such that even where the aims of the study diverge from the main focus of this review, if a ‘nugget’ of evidence relevant to the review questions is provided, this article is included. AND It is assessed to go beyond a superficial description or commentary, i.e. is a competent attempt at research, enquiry, investigation or study (Curnock et al., 2012).This can include qualitative studies using key informant interviews and policy document reviews, surveys, expert legal analyses, case studies, reviews of primary research (if the method was stated) or descriptive models/frameworks (if based on primary data). Exclude the study if: The focus is on agricultural policy, food safety, genetically modified foods and labelling or biotechnology. It analyses trade and investment agreements, WTO disputes but do not also explicitly analyse the impacts (or potential impacts) on health policy processes (prospectively or retrospectively) OR policy space It examines how trade liberalization impacted on health determinants and outcomes but not on health policy processes. Books and book chapters.

#### Selection and appraisal of documents

Electronic searches yielded 1585 results. An additional 51 items were identified through bibliography searches, citation tracking and searches of Google/Google Scholar and institutional websites. After duplicates were removed, 991 unique items remained. Given the realist approach and the limited literature, an intentionally inclusive approach was taken throughout the selection process.

In a preliminary screening, articles were selected based on the test for inclusion derived from realist principles (Table 1), as judged by the titles and abstracts. Commentaries (unless based on empirical evidence or providing key anecdotal evidence), editorials, opinion pieces, conference abstracts and data-free models/frameworks were excluded. After a scoping of included literature, the review scope was narrowed—to ensure sufficiently in-depth analysis could be undertaken—to include just the impact of trade issues (excluding investment) on the three policy areas. With this limitation applied, the first reviewer’s screen resulted in 174 texts being retained for full-text review. A second reviewer screened 10% of all references resulting in 2% differences in opinion regarding evidential relevance or study quality. Given discrepancies were below 10%, after resolving these differences via discussion, the remaining publications were single-screened.

**Figure 2:**
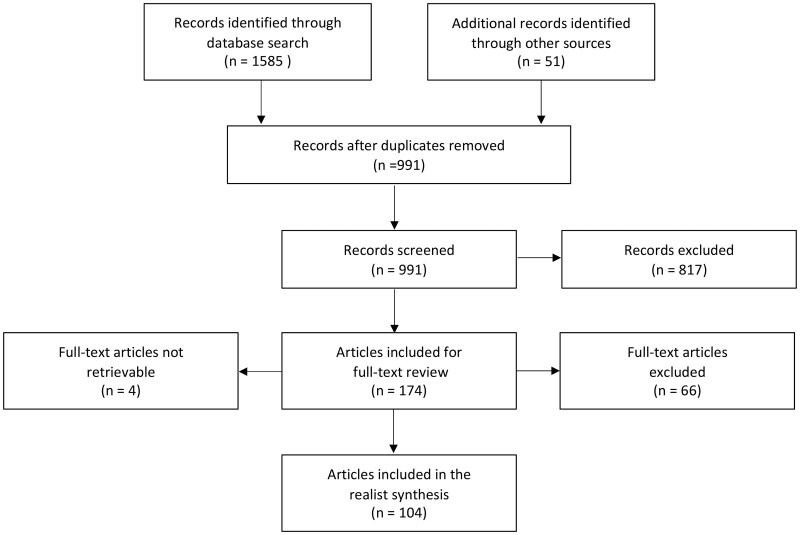
Screening flow diagram.

Full texts were retrieved for 170 of the 174 articles included after initial screening with four articles not retrievable. The 170 full texts were again assessed for relevance based on the test for inclusion. Full-text review resulted in exclusion of a further 66 articles bringing the final number of relevant articles to 104 (Figure 2). Ten per cent of the full texts were again reviewed by the second reviewer resulting in 100% inter-reviewer agreement. The remaining texts were assessed for inclusion by the first reviewer only.

A screening tool (Supplementary Text SII and SIIb) was used to document the rationale for final inclusion/exclusion in the realist synthesis. This included a set of queries regarding study relevance and reliability based on the test for inclusion adapted from a similar set of constructs (Williams *et al.*, 2016). The final 104 articles included in the synthesis were imported into NVivo and stored as individual ‘sources’. Given the diversity of included articles in terms of discipline and methods, it was not possible to apply a single recognized quality appraisal assessment tool to report on overall quality of the studies included in the review. Instead, the realist approach was taken by which each entire study was not assessed for quality but rather each nugget of relevant evidence identified within a primary study was judged on its reliability and relevance to theory development.

## Data extraction, analysis and synthesis processes

Within NVivo, ‘nodes’ were generated for each preliminary explanatory theory. The first reviewer extracted data from each included article that was considered relevant and useful to theory development, including data that supported or challenged each explanatory mechanism and the associated outcomes as well as relevant contextual factors. As additional useful theories were identified new nodes were generated and relevant data extracted. In addition, information on study characteristics (e.g. type of study, methodological approach, health issues covered) was recorded on the screening tool. NVivo was used not only to improve robustness of data analysis but also to improve transparency by providing an audit of the data analysis process. The data extracted under each node were imported into a Word document for analysis. They were analysed and synthesized using a realist approach that was both deductive and inductive. The findings are presented in a narrative synthesis.

## Results

The 104 studies included in the review were from a variety of fields including public health, international law and political science. Accordingly, studies varied in design and methods including prospective analyses of trade and investment agreement texts, analyses of WTO committee meeting minutes and WTO disputes; surveys and key informant interviews; and critical analyses of industry and policy documents. Given that our review question requires investigation of policy decisions but particularly *non-decisions* and the role of power in these outcomes, we identified very few quantitative analyses for inclusion. Furthermore, our enquiry is inherently multi-disciplinary in nature with legal, political and other social science research providing valuable insights. For these reasons, we opted to include analyses based on expert opinion and deductive reasoning, not only empirical research. In most studies, formal power analysis was lacking or limited and understanding contextual elements was generally not included as a primary research objective and typically only discussed superficially.

The analysis presents the evidence for each explanatory theory/mechanism under theory areas based on the three power types outlined in the conceptual framework (Figure 1).

### Instrumental power

Economic liberalization has facilitated increases in efficiency, profitability and global reach of THCCs (Friel *et al.*, 2013, 2015; Appau *et al.*, 2017). As regulation increases and risk commodity consumption declines in High income countries (HICs), THCCs have responded by focusing on developing markets in LMICs (WHO, 2018). As such, THCCs are increasingly interested in influencing domestic risk commodity regulatory environments in LMICs, as well as international rule-setting bodies including the WTO and WHO. As TRCCs grow in size and profitability (Moodie *et al.*, 2013), their capacity to fund ongoing intensive multi-level lobbying strategies gives them a powerful advantage over public health and civil society actors (Fuchs and Lederer, 2007). Lobbying activities occur in both open and increasingly closed spaces as THCCs are granted privileged access to political decision-makers due to concerns about economic growth and the increasing complexity of policy issues (Fuchs and Lederer, 2007). International trade rules provide a valuable legal instrument for THCCs to influence health policy decisions. As a result of these processes, it can be suggested that less powerful health policy actors may voluntarily decide not to act or be forced to make involuntary non-decisions relating to risk commodity regulations.

A number of studies provided evidence of THCC lobbying across multiple trade and health political fora. For example, during China’s WTO accession negotiations British American Tobacco (BAT) intensively lobbied the UK, EU and US officials to petition for among other things, lower tariffs on tobacco products and no restrictions on tobacco advertising (Holden *et al.*, 2010). The alcohol industry has similarly lobbied for favourable trade arrangements (Zeigler, 2009). More recently, during the Trans-Pacific Partnership Agreement (TPPA) negotiations ultra-processed food and beverage corporations undertook extensive lobbying advocating for increased market access, greater regulatory harmonization and investment protections, each with possible implications for nutrition policy space (Friel *et al.*, 2016). THCC’s also use various lobbying tactics to influence the development of international health governance instruments. For example, during negotiations for the Framework Convention on Tobacco Control (FCTC) BAT lobbied the WTO to ensure tobacco was not excluded from multilateral trade agreements (Mamudu *et al.*, 2011).

Decision-makers can be motivated to grant certain business actors privileged access to decision-making spaces given the complexity of trade rules and concerns for economic growth. During both the TPPA and Transatlantic Trade and Investment Partnership agreement (TTIP) negotiations e.g. tobacco companies met privately with US and European Commission (EC) trade officials to discuss the proposed agreements (Crosbie and Glantz, 2014). A Canadian case-study evidenced a close relationship between industry and the trade ministry with one interviewee indicating that the trade ministry was ‘effectively an internal lobby for business’ (Van Harten and Scott, 2016b). A New Zealand study found that the food and beverage industry had a ‘high relative capacity to directly access decision-makers’ in relation to obesity and diabetes policy, as compared to other actor groups (de Bruin *et al.*, 2018).

However, as their legitimacy declines, THCC’s access to certain decision-making spaces can diminish (Hawkins *et al.*, 2019). This may prompt THCCs to engage in ‘venue shifting’—a strategy to claim alternative spaces of influence through shifting decision-making power to fora, in this case legal, including international trade venues, where their interests may be prioritized (Eckhardt *et al.*, 2016; Hawkins *et al.*, 2019). Various studies provide insight into the potential for international trade rules to be used by THCCs and their patron states to directly obstruct, delay or divert resources from progressive public health policymaking. These include WTO rules but also ‘WTO-plus’ rules, deeper than minimum WTO obligations (Bacchetta *et al.*, 2011; Baldwin, 2011; WTO, 2013) and ‘WTO-extra’ rules that extend further behind national borders to reduce what are considered to be non-tariff barriers to trade (Baldwin, 2011). While a detailed review of this literature is included in Supplementary Text SIII, Table 2 summarizes the key mechanisms by which trade rules may provide opportunities for THCCs to influence public health policy decisions as previously described by Kelsey (Kelsey, 2013).

**Table 2:** Key mechanism by which trade rules may limit public health policy space and provide opportunities for TRCCs and their patron states to influence public health policymaking (Kelsey, 2013)

Substantive rules [e.g. in Technical Barriers to Trade (TBT) chapters] Criteria applied to decision-making and choosing between policy options e.g. fulfilling requirements of the ‘necessity test’ (discussed below) Processes to be used in making decisions e.g. pro-business regulatory impact assessments (this may increase TRCC’s *structural power*) Required evidential basis for policy decisions to justify any measure considered trade restrictive under international agreements Documentation, disclosure and reporting requirements for new regulations/policy Obligatory engagement with TRCCs during policymaking processes (this may also increase TRCC’s *structural power*)

While THCCs cannot themselves bring claims against governments at WTO for violating international trade obligations, there is evidence that corporations use international trade-related legal threats in an attempt to force involuntary public health policy non-decisions and prevent policy transfer regionally or globally, especially for tobacco control (Freeman *et al.*, 2008). For example, in the 1990s tobacco companies claimed Thailand’s proposed cigarette ingredients disclosure legislation violated the TRIPS Agreement and Canada and Australia’s proposed plain packaging violated intellectual property rights under TRIPs and NAFTA (Crosbie and Glantz, 2014). More recently, at least four African countries have received warnings from the tobacco industry that their proposed tobacco laws violate international trade and investment agreements (Hawkins and Holden, 2016). At the supra-national level, tobacco companies commissioned a number of legal analyses supporting their argument that the FCTC created both jurisdictional and substantive conflicts with international trade agreements (Weishaar *et al.*, 2012).

When necessary, the alcohol industry is also adopting similar strategies. For example, the alcohol industry threatened a WTO dispute against Thailand if it adopted a proposed ban on alcohol advertising (Casswell and Thamarangsi, 2009) and argued that the Scottish government’s legislation on minimum unit pricing of alcohol is a technical barrier to trade (Weiss, 2015). In Canada’s Yukon Territory, the alcohol industry prevented adoption of specific health warning labels from bottles and cans by arguing the regulation would be in violation of a range of laws including international trade law (O’Brien *et al.*, 2018).

Trade-related legal threats may be effective tools for THCCs to drive involuntary non-decisions by governments due to the complexity of establishing an adequate defence in a WTO dispute and the vagueness of WTO rules (Stumberg, 2013). First, a defending government must convince the dispute panel that their measure passes a ‘necessity test’. This involves a complex multi-step process of proving that the measure is necessary to protect public health in relation to its effect on trade; effective in achieving a specific health objective; is no more trade restrictive than necessary; and there is no less trade-restrictive alternative measure available (Stumberg, 2013; Thow *et al.*, 2017a). The level of justification required is reduced if the measure is based on a relevant international standard (Thow *et al.*, 2017a). WTO dispute panels are required to weigh and balance these factors which can make the likely outcome of a dispute difficult to predict (Stumberg, 2013).

Passing the necessity test is particularly challenging and complex due to significant uncertainty regarding evidential requirements to prove the necessity of a health measure. For example, the SPS Agreement states a measure must be ‘based on’ scientific principles, evidence and risk assessment which leaves some scope for interpretation. Furthermore, it may not be possible for a country to produce indisputable scientific evidence of effectiveness (Stumberg, 2013), particularly for a novel or pre-emptive policy attempting to mitigate a developing threat. For example, a number of countries opposing Brazil and Canada’s ban on tobacco additives and Ireland’s proposed plain packaging asserted there was no scientific evidence that these novel measures would effectively reduce smoking (Lencucha *et al.*, 2016). More recent discussions about Thailand’s proposed alcohol health warning labelling indicate WTO may accept health measures without indisputable evidence of effectiveness but which are grounded in existing science (O’Brien and Mitchell, 2018). However, there is concern that newer agreements like the TPPA will set a higher bar for evidential requirements to justify a health measure (Kelsey, 2013; Labonté *et al.*, 2016). Concurrently, it is a recognized strategy of THCCs to generate their own opposing evidence that can confound a dispute panel’s assessment (Stumberg, 2013).

Vagueness in trade agreement text has resulted in variable interpretations and rulings by dispute panels creating uncertainty when governments evaluate the risk of future potential WTO disputes in light of a trade-related legal threat (Stumberg, 2013). For example, ‘necessity’ was interpreted narrowly in the 1990 case over Thailand’s ban on tobacco imports where it was ruled insufficient justification was provided for the ban as part of a comprehensive tobacco policy. Thailand was forced to reverse the ban and reduce tobacco excise duties (Stumberg, 2013). Similarly, in the 1997 US–Gasoline case, it was ruled that the overall impact of the whole clean air policy could not justify individual provisions within it (Stumberg, 2013). In 2011, Samoa reversed a ban on a fatty meat cut after WTO members ‘questioned the prohibition of a single food item in order to address the […] complex problem of obesity’ (Thow *et al.*, 2017b; World Trade Organization, 2011). In the 2007 Brazil–Tyres case, however, necessity was interpreted progressively and the cumulative contribution of individual measures within a comprehensive approach was accepted (Stumberg, 2013). While there has arguably been a shift towards more progressive interpretations of necessity by WTO panels (Drope and Lencucha, 2014), overall interpretation variability and a lack of case law for alcohol or food policy may still create significant uncertainty of outcome for governments.

If the significant hurdle of proving necessity is passed, a government must establish that their proposed measure is not unjustifiably discriminatory between countries (Stumberg, 2013; Lester, 2015). Satisfying this requirement, however, by applying a measure in a non-discriminatory manner may often not be politically feasible since most public health policy is the result of stakeholder bargaining (von Tigerstrom, 2013, Gruszczynski, 2013). Furthermore, there is no consistent approach regarding what constitutes ‘like’ products when assessing for discrimination between countries (Weiss, 2015).

While some anecdotal evidence exists, empirical evidence that THCCs can effectively promote non-decisions by health departments by generating real or perceived risk of a WTO dispute is, so far, limited. A 2014 Canadian case study found that particularly senior health and safety regulators were concerned with avoiding WTO disputes, although it was not generally reported as a key concern (Côté, 2014). The study also reported that trade disputes were not a primary concern of tobacco control regulators globally, although those considering plain packaging were concerned about the risk of violating intellectual property laws and potential WTO litigation and had adopted a ‘wait and see’ approach to Australia’s WTO plain packaging dispute (Côté, 2014). Another 2016 Canadian case study reported that ministries had changed their decision-making to account for trade concerns, including but not limited to investment arbitration (Van Harten and Scott, 2016a). A 2017 Brazilian case study found that most government stakeholders did not consider trade agreements to pose a threat to tobacco control in Brazil (Drope *et al.*, 2017).

### Structural power

With the majority of modern economies structured along neoliberal lines to facilitate free market competition (Springer *et al.*, 2016; Lencucha and Thow, 2019), political elites are dependent on private sector profitability to achieve set goals of job creation and economic growth (Fuchs and Lederer, 2007). As such, institutional structures and practices may be reoriented to include private actors and prioritize their interests in both national and international decision-making spaces. Within these otherwise closed spaces, THCCs may have significant power to control the policy agenda and shape the rules. While it is challenging to quantify particularly the agenda-setting power of corporations (Fuchs and Lederer, 2007), we did find evidence of institutionalization of industry involvement in policy processes.

Within international public health regulatory and norm-setting bodies, alcohol and food corporations are increasingly privileged with high levels of participation (George, 2018). For example, at Codex meetings where food standards are developed by the Codex Alimentarius Commission, national delegations increasingly consist of industry representatives, leading to concern that the Codex agenda and standards are heavily influenced by private industry (George, 2018). High-income country negotiating position on the UN’s Political Declaration on the Prevention of NCDs was heavily influenced by the food and alcohol industries (Stuckler *et al.*, 2011) and WHO’s associated Global Strategy on Diet, Physical Activity and Health (2004) openly commits the WHO to collaborate with the private sector. Furthermore, the WHO Global Action Plan for the Prevention and Control of NCDs encourages governments to consult with industry on policies and build partnerships with industry to strengthen implementation of NCD prevention measures (George, 2018). Given that international health guidelines and frameworks (such as those mentioned) heavily influence national health policy agendas, by influencing at the international level, THCCs also indirectly shape domestic health policy agendas and policy choices.

There is also substantial evidence that neoliberal political values are deeply embedded in trade institutional arrangements at both national and international levels (John, 1998; Rushton and Williams, 2012; Lencucha and Thow, 2019). As such, formal trade policy structures and practices institutionalize the participation of private actors in policymaking spaces. For example, consultation with private industry in the development of trade proposals is required by law in the USA (Zeigler, 2009). During the TPPA negotiations, 85% of the US trade advisory committee members were private industry and trade group representatives (McNeill *et al.*, 2017a). Analysis of tobacco industry documents indicates high levels of co-operation between the US government and industry in efforts to gain greater access to foreign tobacco markets (Holden *et al.*, 2010). The EC’s 14-member advisory group of experts advising TTIP negotiators included at least seven representatives from various industries, and just one representative from a public health organization (McNeill *et al.*, 2017a). Such frequent liaisons allow close relationships to develop between industry and government such that a revolving door between government and industry is acceptable and an effective strategy for industry to gain privileged access to closed decision-making spaces.

Conversely, public health actors are not generally perceived as legitimate actors within trade institutions and structures and are therefore not invited into otherwise closed and opaque trade policymaking spaces. Without meaningful participation, health actors especially from LMICs are very limited in their capacity to influence domestic or international trade policy (Khan *et al.*, 2015). For example, a health representative sits on just two of the US’ 16 trade policy advisory committees (Lee *et al.*, 2009). An Australian case-study found limited opportunity for civil society or academics to consult on Australia’s overall trade policy or for parliament to consider social impacts/include non-trade objectives in trade agreements (Baker *et al.*, 2019). In a 2018 study, health actors across levels reported being excluded from trade negotiating processes and a lack of consultation to evaluate potential areas of trade and health policy incoherence (Battams and Townsend, 2018). There are some examples of civil society and health actors being invited into domestic trade decision-making spaces through new institutional arrangements, but this does not necessarily result in increased influence (Crosbie *et al.*, 2014). As a formal or *ad hoc* observer on a number of relevant WTO committees, the WHO can contribute to discussions but are not officially permitted to be involved in decision-making (Lee *et al.*, 2009). Furthermore, many LMIC governments may have particularly limited financial, human and technical capacity as well as bargaining power to participate effectively in international trade and relevant health standard-setting spaces (e.g. WTO and Codex) restricting their ability to protect national public health interests (Walls *et al.*, 2015).

The second element of structural power refers to rule-setting power (Fuchs and Lederer, 2007). We identified some evidence that as THCCs seek to grow sales in new markets and governments prioritize export interests, THCCs are increasingly involved in domestic health policy decisions. This reflects the view that industry is legitimate collaborators and partners in national health policy decision-making, as indicated by a number of qualitative studies primarily conducted in LMICs (Bakke and Endal, 2010; Mialon *et al.*, 2016; Battams and Townsend, 2018; Oladepo *et al.*, 2018; Thow *et al.*, 2018). Increased industry involvement appears to be linked with the adoption of individual-level health policy instruments with the least impact on industry profitability or alternatively, total policy inaction. A 2009 analysis of draft alcohol policy texts in Uganda, Malawi, Lesotho and Botswana e.g. found that as a result of significant industry input, alcohol policies in all four countries largely reflected industry interests: focusing on the economic benefits of trade in alcohol; taking an individualistic rather than whole-population approach to alcohol harm reduction; emphasizing active participation of alcohol industry in policy formulation and implementation and self-regulation of alcohol marketing (Casswell and Thamarangsi, 2009). In Malawi, the tobacco industry specifically plays a leading role on the National Working Group on Trade Policy and the Private-Public Dialogue Forum and Malawi remains one of the few countries yet to ratify the FCTC (WHO, 2014).

Given their perceived economic contribution and the increasing complexity of trade agreements, governments also widely perceive industry as key partners in developing domestic trade policy. For example, policy and legal documents in both the USA and EU describe business as key partners in shaping national trade negotiation objectives to prevent trade policies that are unfeasible or negatively impact important industries (Jarman, 2017). This suggests THCCs, including tobacco companies, may have significant influence over trade rules. We identified some evidence to support this. For example, Phillip Morris International’s (PMI) request for ‘harmonization of legitimate, science-based regulations’, an investor-state dispute mechanism, and a comprehensive ‘Trade Related Aspects of Intellectual Property Rights (TRIPS)-plus’ chapter within the TPPA (Fooks and Gilmore, 2014) were all included in the US draft of the agreement (Fooks and Gilmore, 2014).

### Discursive power

The neoliberal ideology that open and free competitive markets in all areas of life will achieve economic growth and shared prosperity (Lencucha and Thow, 2019) is central to contemporary global and domestic policymaking processes across sectors and has deeply influenced the way trade and health policy actors think and behave (Rushton and Williams, 2012; Battams and Townsend, 2018). This has included the individualization of disease aetiology, whereby exposure to a limited number of behaviourally defined risk factors is considered personal responsibility, not determined by complex structural and social forces (Glasgow and Schrecker, 2015). Assisted by their perceived legitimacy and high-level access to decision-making spaces, THCCs have effectively propagated neoliberal framings that have helped entrench these restricted ways of interpreting NCD cause and prevention. Consequently, policy space for addressing NCDs has largely been limited to measures that address individual choice (Navarro, 2007; Rushton and Williams, 2012) but do not interfere with the ‘free’ market to trade goods and services within or across borders. Feedback between institutions and dominant neoliberal ideas, values and frames has entrenched ‘trade over health’ policymaking norms over time. As such, norm compliance is not dictated by interests alone but the function of the dynamics of discursive power.

There is evidence that neoliberal ideas have shaped the interpretation of issues at the intersection of trade and health. At the international level, the dominant perception amongst WTO officials included in one study was that international trade is essential for improving global public health without need for consideration of the possible harms (Gopinathan *et al.*, 2018). Similar perceptions were identified in studies of domestic nutrition policy with trade officials understanding NCDs as problems of ‘individual responsibility’ and demand for risk commodities an issue of choice, not a problem of supply facilitated by trade liberalization (Battams and Townsend, 2018; Thow *et al.*, 2018; Baker *et al.*, 2019). In South Africa, dominant policy actors believed economic growth, achieved in part through international trade and investment, would resolve nutrition problems causing NCDs by increasing consumer wealth (Thow *et al.*, 2018). Some LMIC governments also continue to perceive tobacco exports as important for economic growth (Makoka *et al.*, 2017; WHO, 2014). In Malawi, one study found both health and non-health sector actors perceived tobacco as important for economic stability, job creation and to support health system and service strengthening (Lencucha *et al.*, 2018).

Within this context, where the dominant understanding of NCD causation is congruent with neoliberal assumptions, relatively limited psychological boundaries around NCD prevention interventions have been established. Notably, despite frequent recognition of the upstream determinants of NCDs by relatively authoritative political and scientific institutions, policy decisions still tend to ‘drift’ downstream to those safely within these narrow boundaries (Glasgow and Schrecker, 2015). Specifically, in relation to risk commodities, conceivable options tend to consist largely of demand-side interventions while policies that address system and supply-side issues generally fall outside of policy actors’ ideational boundaries. For example, in both Australia and South Africa nutrition has generally not been considered as a trade policy issue (Battams and Townsend, 2018; Thow *et al.*, 2018; Baker *et al.*, 2019) and global NCD policy recommendations are broadly limited to individualized policy solutions (Glasgow and Schrecker, 2015).

As a result of these described processes, policymaking norms have emerged characterized by a persistent tendency for economic and trade objectives to be prioritized over health resulting in voluntary public health policy inaction. At the supranational level, the WHO’s 2004 Global Strategy on Diet, Physical Activity and Health states that no provision within it should be considered justification for trade-restrictive measures, and important trade issues were left out of the 2011 Political Declaration on the Prevention and Control of NCDs (UNPDNCD) after opposition by the USA and the EU (Orbinski *et al.*, 2011). The FCTC process also reflected the dominance of trade interests in policy decisions. Despite efforts by a number of countries to ensure the FCTC emphasized the priority of public health over international trade and investment objectives, the FCTC remains subordinate to WTO (Mamudu *et al.*, 2011; Gruszczynski, 2015, 2017b).

At the domestic level, policy actors in Australia and Malaysia identified that export interests were often privileged over health objectives (Battams and Townsend, 2018). A study in Kenya, Zambia and Malawi found that even health actors deferred to the ‘dominant economic development norm’ that tobacco is an economic commodity to be promoted (Lencucha *et al.*, 2018), The Fijian Ministry of Health opted for a voluntary over mandatory front-of-package food labelling scheme due to concerns that mandatory labelling would negatively affect trade (Mialon *et al.*, 2016). Tonga is reported to not have proceeded with a proposed restriction on a fatty meat cut, concerned it would interfere with Tonga’s accession to the WTO (Thow *et al.*, 2010). Canadian policymakers involved in health and safety regulatory development were reported to internalize trade norms through ‘regulatory impact assessments’ which include consideration of trade implications for any new regulation, and efforts by policymakers to avoid obstructing the free flow of commercial goods/investment during policy design (Côté, 2014). THCC’s perceived contribution to the economic growth objective is widely argued to prevent governments from regulating risk commodities in an effort to contain industry costs (Mialon *et al.*, 2016; Battams and Townsend, 2018; Hawkins *et al.*, 2018; Thow *et al.*, 2018; Baker *et al.*, 2019).

The dogma that exporting risk commodity industries are essential for economic growth and job creation can also compel governments to pursue the interests of THCCs in trade agreement negotiations and at WTO (Curran and Eckhardt, 2017; Barlow *et al.*, 2018). The USA has threatened trade sanctions against at least five Asian countries if they did not open their markets to foreign tobacco products (Charoenca *et al.*, 2012; Mackenzie and Collin, 2012) and nearly all trade and investment agreements negotiated by the USA eliminate or reduce their trading partners’ tobacco tariffs (Labonte *et al.*, 2011). In 2014, when Jamaica and Ireland were developing tobacco control legislation, the USA claimed the measures would contravene intellectual property obligations under international trade and investment agreements (Gilmore *et al.*, 2015). As recently as 2018, the EU, USA and UK supported tobacco companies to oppose cigarette ingredients disclosure in Thailand at the WTO (Charoenca *et al.*, 2012).

As their legitimacy declines in HICs, tobacco companies have turned to more economically vulnerable LMICs to act on their behalf. LMICs have been encouraged to use WTO forums to make an economic development argument against tobacco control by raising Article 12.3 of the Technical Barriers to Trade (TBT) Agreement that requires the special needs of developing countries to be taken into account. This was used to oppose Canada’s ban on tobacco additives to help mitigate youth smoking, the European Union Tobacco Products Directive (EUTPD), Brazil’s additives ban and Australia’s plain packaging regulation (Lencucha *et al.*, 2016; Gruszczynski). Five LMICs were supported by the tobacco industry to mount the 2014 WTO challenge against Australia’s plain packaging (Curran and Eckhardt, 2017). The tobacco industry also supported Malawi to raise a trade concern at the TBT committee meeting over Canada’s Cracking Down on Tobacco Marketing Aimed at Youth Act and Brazil’s ban on flavoured cigarettes (Lencucha *et al.*, 2018). At the supranational level, a number of member states strongly opposed including a recommendation to ban slim cigarettes in the FCTC policy guidelines based on an economic rationale (Gruszczynski, 2017a).

The same trade and economic rationale have compelled governments to pursue the interests of the processed food and alcohol industries within WTO forums. Concerns have been raised at WTO’s TBT Committee in the interest of the processed food industry including over Peru, Chile and Thailand’s proposed food labelling regulations (Thow *et al.*, 2017a; Barlow *et al.*, 2018). After the EU and USA complained that Colombia’s mandatory alcohol health warning labelling regulation was overly burdensome and costly to trade, Colombia reduced the range of alcohols covered by the policy and made regulatory compliance voluntary (Barlow *et al.*, 2018).

THCCs encourage the trade over health policy norm by using issue framing that resonates with accepted neoliberal logic, goals and values. Industry has widely used generic economic arguments that THCCs are vital for revenue and job creation (Gilmore *et al.*, 2015). THCCs have also applied specifically trade-focused economic framing to argue against progressive tobacco policy including in New Zealand (Kelsey, 2017), Australia and the UK (Crosbie *et al.*, 2018; Mackenzie *et al.*, 2018). The food industry in Fiji has persistently argued that additional health-protective food policies would have a significant negative trade/economic impact and make Fiji uncompetitive internationally (Mialon *et al.*, 2016).

THCCs also use trade rules to shift public and political discourse, from health to a legal/technical focus (Curran and Eckhardt, 2017). The tobacco industry widely claimed that Australia’s tobacco plain packaging legislation was in violation of international intellectual property rules despite consistent legal advice to the contrary (Freeman *et al.*, 2008; Crosbie and Glantz, 2014). This suggests the tactic was intended to create an alternative discursive reality (Crosbie and Glantz, 2014; Ulucanlar *et al.*, 2016; Mackenzie *et al.*, 2018) with the purpose of chilling regulatory progress and not necessarily to pursue and win a legal case. Lastly, tobacco companies have also capitalized on neoliberal values claiming that tobacco control is government overreach and threatens individual freedom of choice including in Australia, the UK and Canada (Baker *et al.*, 2017; Mackenzie *et al.*, 2018). Along similar lines, the alcohol industry has widely and persistently propagated an individual-level framing of alcohol-related harm (Mccambridge *et al.*, 2018).

## Discussion

This review identifies evidence of THCCs exercising and likely benefiting from each of the three key forms of power outlined in our conceptual framework expressed via various mechanisms. This power resides at both the national and international level and in spaces often closed to health and civil society actors but into which THCCs have been invited. The often hidden and invisible nature of power and non-decisions makes empirical analyses and drawing causal inference between processes of power and outcomes inherently very challenging. However, our findings do indicate linkages between power exercised by THCCs and public health ‘non-decisions’. The framework also provides initial insights into how proposed strategies for change might effectively challenge existing power relations.

Firstly, evidence indicates THCCs exercise instrumental power through their relationships (direct lobbying of trade policymakers) and rules (threats of trade rule violations or operating through governments to access legal mechanisms). Challenging industry’s instrumental power over trade policy might include bans on both THCC political funding and lobbying itself as well as closing the revolving door between government and industry. However, such strategies remain largely unexplored in the trade and health literature. Strengthening countervailing public health lobbying will be challenging given existing money and resource imbalances.

Post-treaty implementation measures to defend health policy space and minimize the impact of trade-related arguments, legal threats and challenges include strengthening public health coalitions (Drope and Lencucha, 2014). For example, developing a multi-sectoral coalition and long-term relationship-building with trade officials meant Australian health actors were trusted to provide sound legal advice to government about the legality of standardized packaging (Crosbie *et al.*, 2018). To counter industry legal threats broad international issue networks advocating for Canada and Brazil’s tobacco additives bans were also established (Drope and Lencucha, 2014).

Other strategies to increase government confidence and ability to design policies that are consistent with trade rules include capacity building within national health departments on trade issues through technical training and cross-departmental collaboration (Thow *et al.*, 2010; Drope and Lencucha, 2013; 2014). Close co-ordination between health and trade officials was observed in both Canada and Brazil when developing their tobacco additives bans to ensure compliance with trade law and pre-empt opposition (Drope and Lencucha, 2014). Neither case proceeded to a WTO dispute. Similarly, in Australia close co-ordination between health and trade officials was essential both in building cross-sectoral support for standardized packaging and for developing a sound legal argument to defend against industry threats and in the eventual WTO (and investment) litigation (Drope and Lencucha, 2014; Crosbie *et al.*, 2018).

In relation to international trade rules themselves, some experts have recently argued that the relevant WTO Agreements do in fact give governments significant space to design and implement, particularly tobacco control measures, but possibly also alcohol and food regulation, provided they are supported by evidence and are non-discriminatory (Drope and Lencucha, 2013; Voon, 2013; Drope and Lencucha, 2014; Thow *et al.*, 2017a). While supporting evidence is primarily drawn from tobacco control-related WTO case law, there may also be some relevance of these arguments for carefully crafted food and alcohol regulations. Analyses of TBT Committee meeting minutes covering trade concerns raised over labelling regulations of processed foods and alcohol health warning labelling tentatively support this (Thow *et al.*, 2017a; O’Brien and Mitchell, 2018). In Table 3, we present a summary of key conditions that, if met, may reduce the scope for THCCs or other governments to use trade rules as a tool for preventing tobacco, alcohol and food regulation.

**Table 3:** Conditions that may reduce restrictions on tobacco, alcohol and nutrition policy space created by international trade rules

Use by public health advocates of language familiar to trade practitioners (Drope and Lencucha, 2014) Clear attempt to integrate health and trade objectives rather than reject principles of free trade outright (Drope and Lencucha, 2014) Strong invocation of parties’ legal commitments to international health agreements (e.g. FCTC) or compliance with international standards (Stumberg, 2013; von Tigerstrom, 2013; Drope and Lencucha, 2014; Russell *et al.*, 2014) Sufficient evidence to support the legitimacy, effectiveness and necessity of the measure to achieve a specific health outcome. It may be acceptable that evidence is in the form of quantitative projections or qualitative reasoning (Stumberg, 2013) Consistent reiteration of the importance of the health objective (Drope and Lencucha, 2013; Drope and Lencucha, 2014) Emphasis the policy is a necessary part of a mutually supportive comprehensive set of measures, meaning that adopting one measure is not an alternative to other complementary measures (Thow *et al.*, 2017; World Health Organisation Framework Convention on Tobacco Control and McCabe Centre for Law and Cancer, 2019) Policies are designed to be as least trade restrictive as necessary without compromising elements essential to the measures effectiveness (Thow *et al.*, 2017) Policies are designed so as not to discriminate between similar imported and domestic products with clear argument for why the products have different end uses and physical characteristics. For example, a challenge that a labelling requirement for only certain types of calorie dense, low nutrition snack is discriminatory against certain imported foods, could be argued against by outlining these snack foods are not like products under the TBT to nutritious foods consumed at mealtimes (von Tigerstrom, 2013).

It is important to note, however, that satisfying these conditions may not protect novel measures, particularly those not supported by an international convention or set of standards. They do not take into account the politics of policymaking that very often demands compromise resulting in regulations that may discriminate between like products from different countries (von Tigerstrom, 2013). They also may not protect supply-side measures which may be highly trade restrictive by design, e.g. product bans. Furthermore, having to satisfy these measures may be challenging for some developing countries with limited legal/technical resources to design policy to meet these conditions, the capacity to conduct their own research, or present a comprehensive defence in trade fora. Finally, more recently negotiated regional trade and investment agreements, like the TPPA and TTIP may establish higher bars for meeting some of these conditions, in particular higher evidential requirements (Kelsey, 2013).

Secondly, neoliberal-oriented institutional structures, practices and goals mean THCCs are often granted privileged access to trade and health decision-making spaces where their interests limit the scope of the agenda. Mobilizing broad coalitions to claim greater access to trade policy decision-making spaces and increase the visibility and legitimacy of health interests on the agenda will be important to challenge structural power. For example, in Australia, a broad network of tobacco control advocates managed to gain legitimacy within trade policy spaces while the absence of such a network mobilized on unhealthy diets and nutrition was an impediment to generating attention to this issue in Australian trade policy (Baker *et al.*, 2019). A strong domestic issue network developed in support of Thailand’s graphic warning label regulation for tobacco products was pursued despite subsequent industry legal threats (Drope and Chavez, 2015).

Limiting industry representation on government trade committees as well as strengthening government institutional capacity for healthy trade policy will also be important to challenge THCC structural power. At the national level, Thailand is often cited as an example of how sustained investment in technical capacity building and inter-departmental co-ordination between trade and health agencies can generate a common understanding of key health and trade policy issues and bring health actors and considerations into trade policy negotiating forums (Thaiprayoon and Smith, 2015; Thow *et al.*, 2017b). Importantly, however, it is uncertain whether these strategies significantly changed Thailand’s trade negotiating position highlighting the importance of exposing and challenging power in all its forms. These strategies may have contributed to the health agency’s confidence to pursue tobacco control regulation including a graphic health warning labelling system, despite trade-related threats from industry (Drope and Chavez, 2015). Strengthened global institutional capacity will also be important to strengthen attention to health interests in international trade policy including through stronger WHO leadership and engagement on health issues at the WTO; and providing technical assistance to governments to more effectively assert health goals in trade policy at the national level (Smith *et al.*, 2009; Baker *et al.*, 2015).

Addressing industry structural power in relation to domestic health policy and international health standards, norms and laws will require structures and rules governing interactions between THCCs and both governments and international public health standard-setting bodies (George, 2018). For example, the FCTC legally obligates parties under Article 5.3 to adopt measures that protect ‘their public health policies related to tobacco control from commercial and other vested interests of the tobacco industry’ (WHO, 2008). However, there has been selective and incomplete implementation of recommended measures allowing significant ongoing opportunities for industry policy influence, again indicating other forms of power are at play (Fooks *et al.*, 2017).

Third, our findings suggest THCCs attempt to exercise agency over discursive power through reinforcing various framings of health issues in ways that resonate with neoliberal logic and values. While it is impossible to draw causal inference, there was evidence that decision-makers’ individualized interpretation of health issues, the boundaries around acceptable solutions and resulting dominant policy norms of ‘trade over health’ aligned with industry framings.

Counteracting these processes include amplifying and propagating alternative framings of trade and health issues. For example, in Australia, tobacco control advocates focused on framing standardized packaging around the direct harms of tobacco and Australia’s commitment to the FCTC and exposing the manipulative nature of the industry’s previous legal attacks (Crosbie *et al.*, 2018). They also successfully built understanding amongst trade actors of standardized packaging not as a trade barrier, but as contributing to economic prosperity, health and well-being (Crosbie *et al.*, 2018). Due to issue complexity, engaging the public and political leaders on trade and nutrition or alcohol issues will, however, be more challenging. Health advocates will likely need to develop simple frames that emphasise the direct and immediate impacts of trade agreements on nutrition (Baker *et al.*, 2019) or alcohol-related harm. This will be important to encourage the understanding of NCDs and risk commodity consumption as system-level problems helping to expand the range of acceptable policy solutions.

International health instruments including standards, guidelines and particularly legally binding agreements can also contribute to shifting policy norms and increase governments’ confidence in adopting health measures despite trade-related concerns or legal threats (von Tigerstrom, 2013; Barlow *et al.*, 2018). Given they provide evidence of effectiveness and to some extent indicate necessity, international health instruments can also support the assertion of health objectives more strongly in WTO fora (Lester, 2015; Gruszczynski, 2017b). Brazilian health policy actors have reported confidence in their right to regulate tobacco in a manner consistent with the FCTC (Drope *et al.*, 2017) and relied heavily on the FCTC in its defence of a ban on cigarette flavouring and additives (Drope and Lencucha, 2014). Australia also drew on the FCTC in its WTO defence over plain packaging (Drope and Lencucha, 2014; Crosbie *et al.*, 2018).

While through their discursive power, THCCs can foster and reinforce neoliberal framings and norms, our findings suggest the pervasive individualistic interpretation of NCDs, limited scope of solutions and ‘trade over health’ policy norms cannot be explained by TRCC agency alone. Rather, our findings tend to support the ‘structuration perspective’ that discursive power is also generated from socio-political systems (Giddens, 1984; Fuchs and Lederer, 2007) and the system theorists’ view that system structures and goals are strongly, although variably, determined by a dominant neoliberal paradigm (Meadows, 2008). Furthermore, there is a duality to the neoliberal system in that while policy actors can shape it they are also enabled and constrained by it (Giddens, 1984), including in relation to exercising or challenging discursive power but also, we suggest, other forms of power too.

We argue therefore that adopting the strategies to challenge THCC power described so far, as well as their ultimate effectiveness, will likely be limited under the constraints of an overarching neoliberal paradigm and system. As such, our analysis indicates that sustainably transforming existing power relations that drive health policy non-decisions will also likely require the development and adoption of a new paradigm with public interest and sustainability values and goals, supporting similar recent calls from public health academics (Schram and Goldman, 2020). While hugely ambitious, the COVID-19 pandemic and broader climate crisis may offer a rare window of opportunity for public health actors to work with social, environmental, and new economics advocates and build support for such an alternative political and economic paradigm. The basis of such models already exist in indigenous communities and at grassroots level in the GlobalSouth and these alternative knowledge and value systems should be centred in the development of an alternative approach (Droz, 2019; Jones, 2019). In Europe. Raworth’s Doughnut Economics model that replaces the primary goal of economic growth with an equity-focused goal of meeting the needs of all within the means of the planet has gained significant interest (Raworth, 2017).

**Figure 3:**
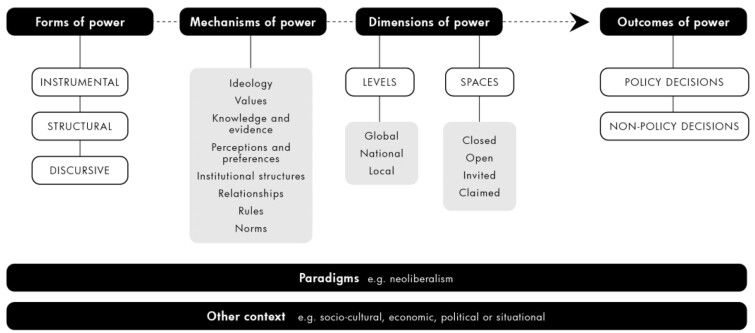
Conceptual framework for analysing power in public health policymaking (revised).

This analysis tentatively supports the potential utility of the conceptual framework developed in this work for power analysis in public health policymaking. The analysis indicates that a possible revision of the conceptual framework to emphasize the broad influence of paradigms at the system level on processes of power may be useful (Figure 3).

## Limitations

This review has a number of key limitations. Given the multi-disciplinary nature of the review topic as well as our restricted capacity to undertake multiple secondary iterative literature searches in keeping with the realist approach, it is possible that relevant explanatory mechanisms and data that supported or challenged them, was not captured in this review. Also, identification of explanatory mechanisms may have been limited due to the very few studies identified on trade and health policy that explicitly engaged with theories of power.

## Conclusions

Exposing all forms of power and their associated mechanisms is essential for identifying and evaluating strategies that can generate the shifts in power required to achieve transformative governance and policy change in health, trade and other sectors for tackling NCDs. However, theoretical and empirical research examining power at the nexus of trade and health policymaking, and in health policy analysis more broadly, is currently very limited. More rigorously incorporating theories of power in health policy analyses would be useful for understanding how to push beyond the individualistic interpretation of NCD risk and outcomes and expand ideational boundaries to include strategies that address health-harmful product supply but also the social and economic conditions within which consuming these commodities occurs.

The findings of this review raise a range of other important research questions including e.g. how do power relations and dynamics between trade and health actors (and their associated outcomes) compare in different contexts e.g. by varying levels of economic development or socio-economic inequality, or under different (and different combinations of) predominant political and economic paradigms? We hope the framework developed in this work is a helpful starting point for shaping a research agenda that covers these and other key questions, as well as providing a useful tool for future analyses of power in health policymaking.

## Supplementary data

Supplementary data are available at *Health Policy and Planning* online.

## Funding

Penelope Milsom received doctoral funding from the Wellcome Trust [grant number 203286/Z/16/Z ] to support this research.

## Supplementary Material

czaa148_Supp
