# Corporate power and the international trade regime as drivers of NCD policy non-decisions: a realist review

**DOI:** 10.1093/heapol/czab013

**Published:** 2021-04-25

**Authors:** Penelope Milsom, Richard Smith, Phillip Baker, Helen Walls

*Health Policy and Planning*, doi: 10.1093/heapol/czaa148.

In the originally published version of this manuscript, there was an error in Figure 1 and Figure 3. The second text box under the parent text box "outcomes of power" should read: "policy non-decisions" instead of "non-policy decisions". This error has now been corrected online.

