# Supplementary material for: Corporate power and the international trade regime preventing progressive policy action on non-communicable diseases: a realist review
Source: Health Policy Plan. 2020 Dec 4;36(4):493–508. doi: 10.1093/heapol/czaa148 (PMC8128013; doi:10.1093/heapol/czaa148)
Supplement: czaa148_Supp [file czaa148_supp.zip › Supplementary Text IIb.docx]

**Supplementary Text IIb:** Populated Screening Tool

| **Author, title of publication (year):** | **Type of study/ source** | **Method stated** | **Data source stated (or referenced)** | **Empirical research** | **Policy area** | **Inclusion/ Exclusion criteria: Relevant** | **Inclusion/ Exclusion criteria: Reliable** | **Decision to include/ exclude?** | **Reason for decision** | **Full text accessible** |
| --- | --- | --- | --- | --- | --- | --- | --- | --- | --- | --- |
| Al Ansari et al, Extent of alcohol prohibition in civil policy in Muslim majority countries: the impact of globalization. (2016) |  |  |  |  |  | RelNo |  | Ex | no relevant discussion about how health related policy-making processes are affected by trade-related issues | Yes |
| Aldis et al, The Trans-Pacific Partnership Agreement: A Test for Health Diplomacy (2013) |  |  |  |  |  | RelNo |  | Ex | no relevant discussion about how health related policy-making processes are affected by trade-related issues | Yes |
| Anderson, Global alcohol policy and the alcohol industry (2019) | Anrev |  |  |  |  | RelNo |  | Ex | No relevant trade-related factors covered | Yes |
| Appau et al, Disentangling regional trade agreements, trade flows and tobacco affordability in sub-Saharan Africa (2017) | AnQuant | 450 H. Jarman | DYes | Yes | PTob | RelYes | QualYes | Inc | Demonstrates how the evolving trade and investment regime enables the productive and structural power of the industry | Yes |
| Assunta and Dorotheo, SEATCA Tobacco Industry Interference Index: a tool for measuring implementation of WHO Framework Convention on Tobacco Control Article 5.3 (2016) | Ananaly |  |  |  |  | RelNo |  | Ex | Measures implementation of the FCTC and documents ongoing methods of industry interference but does not provide any mechanisms relevant to this review. | Yes |
| Assunta et al, Tobacco industry interference: A review of three South East Asian countries (2017) | AnPol |  |  |  |  | RelNo |  | Ex | Discussed industry tactics used to influence tobacco control policy but no discussion of trade and investment-related factors. | Yes |
| Baker et al, Generating political priority for regulatory interventions targeting obesity prevention: An Australian case study (2017) | AnPol | MYes | DYes | Yes | Pmulti | RelYes | QualYes | Inc | Includes mechanisms related to the power of neoliberal ideas | Yes |
| Baker et al, Strengthening Trade and Health Governance Capacities to Address Non-communicable Diseases in Asia: Challenges and Ways Forward (2015) | AnPol | MNo | DYes | No | Pmulti | RelYes | QualYes | Inc | Provides some novel nuggets of evidence on how political norms influence policy decisions and also contextual issues | Yes |
| Baker et al, Trade and nutrition policy coherence: a framing analysis and Australian case study (2019) | AnQuant | MYes | DYes | Yes | PNutr | RelYes | QualYes | Inc | Evidence of framing of NCD issues and how this influences perception of whether issues are relevant to trade policy or not | Yes |
| Baker, Kay and Walls, Trade and investment liberalization and Asia's noncommunicable disease epidemic: a synthesis of data and existing literature (2014) | AnRev | MYes | DYes | No | Pmulti | RelYes | QualYes | Inc | Some relevant nuggets of evidence for policy space and policy chill | Yes |
| Bakke and Endal, Alcohol policies out of context: drinks industry supplanting government role in alcohol policies in sub-Saharan Africa (2009) | AnPol | MYes | DYes | Yes | PAlc | RelYes | QualYes | Inc | Indicates how alcohol industry attempts to prime the policy context of emerging markets | Yes |
| Baldwin et al, 1st Century regionalism: Filling the gap between 21st Century and 20th Century trade rules (2011) | AnLegal | MNo | Dyes | No | Pmulti | RelYes | QualYes | Inc | Provides evidence of the impacts of trade rules, including WTO plus and extra rules on health policy space | Yes |
| Balwicki et al Tobacco industry interference with tobacco control policies in Poland: legal aspects and industry practices (2015) | AnPol | MYes | DYes | Yes | PTob | RelYes | QualYes | Inc | Provides nuggets of evidence of tobacco company use of trade agreements to delay policy, also evidence of tobacco companies using the government in one country to try to influence tobacco control in another country via the WTO | Yes |
| Barlow et al, The health impact of trade and investment agreements: a quantitative systematic review and network co-citation analysis (2017) | AnRev |  |  |  |  | RelNo |  | Ex | Review of impacts of trade agreements on health outcomes, not policy processes | Yes |
| Barlow et al, Trade challenges at the World Trade Organization to national noncommunicable disease prevention policies: a thematic document analysis of trade and health policy space (2018) | AnArb | MYes | Dyes | No | PNutr | RelYes | QualYes | Inc | Provides evidence of how trade challenges at the WTO can restrict policy space and potentially have a regulatory chilling effect | Yes |
| Battams and Townsend, Power asymmetries, policy incoherence and noncommunicable disease control - a qualitative study of policy actor views (2018) | AnQual | MYes | DYes | Yes | Pmulti | RelYes | QualYes | Inc | Provides useful data on how power relations affect trade-sensitive health policy decisions | Yes |
| Baumberg and Anderson, Trade and health: how World Trade Organization (WTO) law affects alcohol and public health (2008) | AnRev | MYes | DYes | No | PAlc | RelYes | QualYes | Inc | provided some relevant nuggets of evidence on misperceptions about the restrictions WTO rules places on health policy | Yes |
| Beard, Trans-Pacific Partnership Agreement and the public health (2015) | AnCom | Mno | Dyes | No | Pmulti | RelYes | QualCT | Ex | Opinion piece providing no novel nuggets of evidence | Yes |
| Bertscher et al, Unpacking policy formulation and industry influence: the case of the draft control of marketing of alcoholic beverages bill in South Africa (2018) | AnPol |  |  |  |  | RelNo |  | Ex | Discussed industry tactics used to influence tobacco control policy but no discussion of trade and investment-related factors. | Yes |
| Birnbaum, Have international trade agreements been good for your health? (2016) | Anrev |  |  |  |  |  |  | FTNA | Full text not available | No |
| Bump, Political economy analysis for tobacco control in low- and middle-income countries (2013) |  |  |  |  |  | RelNo |  | Ex | In this literature review relevant mechanisms are identified from other sources already included in the review | Yes |
| Casswell and Thamarangsi, Reducing harm from alcohol: call to action (2009) | AnCom | MNo | DYes | No | PAlc | RelYes | QualYes | Inc | Although this is an editorial it was judged that the useful nuggets of evidence of trade-related legal threats from industry in relation to alcohol regulations | Yes |
| Casswell, Why do we not see the corporate interests of the alcohol industry as clearly as we see those of the tobacco industry? (2012) | Ananaly |  |  |  |  | RelNo |  | Ex | industry tactics not related to trade and investment | Yes |
| Chalmers et al, Real or perceived impediments to minimum pricing of alcohol in Australia: Public opinion, the industry and the law (2013) | AnCom |  |  |  |  | RelNo |  | Ex | This is a commentary and does not provide relevant nuggets of evidence relating to how alcohol policy is affected by trade-related factors. | Yes |
| Charoenca et al, Success counteracting tobacco company interference in Thailand: An example of FCTC implementation for low- and middle-income countries (2012) | Case study | MYes | DYes | Yes | PTob | RelYes | QualYes | Inc | Provides data for context in which it is possible to prevent TNCs influencing health policies. | Yes |
| Charvel et al, A process to establish nutritional guidelines to address obesity: Lessons from Mexico (2015) | AnPol |  |  |  |  | RelNo |  | Ex | Evidence of industry involvement in nutrition policy development, how they frame the issue, and how economic arguments trump health arguments, although not related to trade. | Yes |
| Collin et al, Government support for alcohol industry: promoting exports, jeopardising global health? (2014) | Ananaly | MNo | DYes | No | PAlc | RelYes | QualYes | Ex | No sufficiently relevant nuggets of evidence | Yes |
| Collins, Tobacco control, global health policy and development: towards policy coherence in global governance (2012) | Ananaly | MNo | DYes | No | PTob | RelYes | QualYes | Inc | Few relevant nuggets of evidence on trade challenges in relation to tobacco control policies | Yes |
| Cotê, A Chilling Effect- The impact of international investment agreements on national regulatory autonomy in the areas of health, safety and the environment (2014) | AnQual | MYes | DYes | Yes | Pmulti | RelYes | QualYes | Inc | Empirical analysis of regulatory chill hypothesis | Yes |
| Crosbie et al, Advancing progressive health policy to reduce NCDs amidst international commercial opposition: Tobacco standardised packaging in Australia (2018) | AnQual | MYes | DYes | Yes | PTob | RelYes | QualYes | Inc | Provides nuggets of evidence relating to strategies to counter industry use of power | Yes |
| Crosbie et al, Defending strong tobacco packaging and labelling regulations in Uruguay: transnational tobacco control network versus Philip Morris International, 2018 | AnArb | MYes | DYes | Yes | PTob | RelYes | QualYes | Inc | Provides valuable evidence of contextual elements necessary to avoid policy chill in a small vulnerable middle-income country | Yes |
| Crosbie et al, Health Pre-emption Behind Closed Doors: Trade Agreements and Fast-Track Authority (2014) | AnCom | MNo | DYes | No | PTob | RelYes | QualCT | Inc | Provides mechanisms for opening of spaces but without necessarily changing power dynamics | Yes |
| Crosbie, Tobacco industry argues domestic trademark laws and international treaties preclude cigarette health warning labels, despite consistent legal advice that the argument is invalid (2014) | AnQual | MYes | DYes | Yes | PTob | RelYes | QualYes | Inc | Analysis of tobacco company documents indicating tobacco company strategies to generate policy chill using WTO agreements (TRIPS, GATT) | Yes |
| Curran and Eckhardt, Smoke Screen? The Globalization of Production, Transnational Lobbying and the International Political Economy of Plain Tobacco Packaging (2017) | Case study | MYes | DYes | Yes | PTob | RelYes | QualYes | Inc | Provides data for theories for how TNCs use WTO dispute forum in an attempt to generate policy chill | Yes |
| Davoren, Legal interventions to reduce alcohol-related cancers (2011) | Ananaly |  |  |  |  | RelNo |  | Ex | No relevant discussion about how health related policy-making processes are affected | Yes |
| de Bruin, Who Are the Key Players Involved with Shaping Public Opinion and Policies on Obesity and Diabetes in New Zealand? (2018) | AnQuant | 449 | DYes | Yes | PNutr | RelYes | QualYes | Inc | Provides nugget of evidence of industry privileged access to health policymakers | Yes |
| De Vogli and Renzetti, The Potential Impact of the Transatlantic Trade and Investment Partnership (TTIP) on public health (2016) | AnTIAp | MNo |  | No | Pmulti | RelYes | QualYes | Inc | Discussed what policy areas the authors assessed to be affected by the TTIP with some explanation of why/how | Yes |
| Diaz-Bonilla, Lost in Translation The Fractured Conversation about Trade and Food Security (2015) |  |  |  |  |  | RelNo |  | Ex | No discussion of how trade and investment-related factors my influence nutrition policy (although does mention agricultural policy but this is outside the scope of this review) | Yes |
| Drope & Chavez, Complexities at the intersection of tobacco control and trade liberalisation: evidence from Southeast Asia (2015) | AnMix | MYes | DYes | Yes | PTob | RelYes | QualYes | Inc | Provides evidence of industry use of trade-related legal threats in relation to tobacco control regulations | Yes |
| Drope and Lencucha, Evolving norms at the intersection of health and trade (2014) | AnArb | MYes | DYes | No | PTob | RelYes | QualYes | Inc | Provides nuggets of evidence relating to how misperceptions of risks associated with TIAs and also factors that contribute to successful introduction of health policy despite trade challenges | Yes |
| Drope and Lencucha, Tobacco control and trade policy: Proactive strategies for integrating policy norms (2012) | Ananaly | Mno | Dyes | No | Ptob | RelYes | QualYes | Inc | Provide nuggets of evidence on strategies to overcome industry influence over health policy | Yes |
| Drope et al, Conceptualizing threats to tobacco control from international economic agreements: the Brazilian experience (2017) | AnQual | MYes | DYes | Yes | PTob | RelYes | QualYes | Inc | Provides nuggets of evidence of Brazilian government confidence to regulate in line with the FCTC despite trade-related legal threats | Yes |
| Eckhardt et al, Tobacco Control and the World Trade Organization: Mapping Member States’ Positions after the Framework Convention on Tobacco Control (2017) | Andescript | MYes | DYes | Yes | PTob | RelYes | QualYes | Inc | provides evidence relating to motivation for countries to bring a WTO claim or raise tobacco policy as an issue of concern to trade | Yes |
| Fooks & Gilmore, International trade law, plain packaging and tobacco industry political activity: the Trans-Pacific Partnership (2014) | AnTIAp | MNo | DYes | No | PTob | RelYes | QualNo | Inc | Provides expert analysis of leaked TPP chapters potential impact on plain packaging | Yes |
| Fooks et al, Controlling corporate influence in health policy making? An assessment of the implementation of Article 5.3 of the World Health Organization framework convention on tobacco control (2016) | AnQuant |  |  |  |  | RelNo |  | Inc | Examines implementation of Article 5.3, strength of parties ‘efforts and how different implementation approaches exposes policy processes to ongoing industry influence. | Yes |
| Freeman et al, The case for the plain packaging of tobacco products (2008) | AnQual | tobacco as a normal good and to seek out “fair and equitable treatment” for | DYes | No | PTob | RelYes | QualYes | Inc | Provides evidence that corporations use international trade-related legal threats in an attempt generate policy chill | Yes |
| Friel et al, A new generation of trade policy: potential risks to diet-related health from the trans pacific partnership agreement (2014) | AnTIAp | MYes | DYes | No | Pmulti | RelYes | QualYes | Ex | Primarily analyses impacts on health but very limited discussion on impacts on health policy processes | Yes |
| Friel et al, Monitoring the impacts of trade agreements on food environments (2015) | AnFram | MYes | DYes | No | PNutr | RelNo | QualCT | Inc | Several mechanisms outlined linking trade and investment regime change to changes in food environments and links to food companies | Yes |
| Friel et al, Shaping the discourse: what has the food industry been lobbying for in the Trans Pacific Partnership trade agreement and what are the implications for dietary health? (2016) | AnQual | MYes | DYes | Yes | PNutr | RelYes | QualYes | Inc | Nuggets of evidence on food industry lobbying in the TPP | Yes |
| Friel et al, Trade Policy and Public Health (2015) | AnRev | MNo | DYes | No | Pmulti | RelYes | QualCT | Inc | Provides evidence that TIAs facilitate expansion of TNCs | Yes |
| Friel, et al. The nexus between international trade, food systems, malnutrition and climate change | AnRev | MNo | DYes | No | PNutr | RelYes | QualYes | Ex | Review providing no new nuggets of evidence | Yes |
| Garton, Systematic Assessment of Policy Space in New Zealand for Priority Food Environment Policies for Obesity/Ncd Prevention in Relation to International Trade and Investment Agreements (2017) |  |  |  |  |  |  |  | FTNA | Full text not available | No |
| George, An Unwelcome Seat at the Table: The Role of Big Food in Public and Private Standard-Setting and Its Implications for NCD Regulation (2018) | AnPol | MNo | DYes | No | PNutr | RelYes | QualYes | Inc | Evidence of industry involvement in nutrition policy standard-setting | Yes |
| George, Not so sweet refrain: sugar-sweetened beverage taxes, industry opposition and harnessing the lessons learned from tobacco control legal challenges (2018) | AnPol | MNo | DYes | No | PNutr | RelYes | QualYes | Inc | Provides nuggets of evidence based on legal analysis of tobacco policy that if countries take certain approaches, there is space for SSB tax without grounds for trade challenge | Yes |
| Gilmore et al, Exposing and addressing tobacco industry conduct in low-income and middle-income countries (2015) | AnRev | MNo | DYes | No | PTob | RelYes | QualYes | Inc | Provides nuggets of evidence relating to industries use of economic framing to argue tobacco companies are important for economic growth and job creation | Yes |
| Gilmore, Understanding the vector in order to plan effective tobacco control policies: an analysis of contemporary tobacco industry materials (2012) | AnQual | Myes | Dyes | Yes | Ptob | RelNo | QualYes | Ex | No sufficiently relevant nuggets of evidence | Yes |
| Glasgow and Schrecker, the double burden of neoliberalism? Noncommunicable disease policies and the global political economy of risk (2015) | Ananaly | Mno | Dyes | No | Pmulti | RelYes | QualYes | Inc | Discusses the individualization of NCD |  |
| Gomes and Lobstein, Food and beverage transnational corporations and nutrition policy (2011) |  |  |  |  |  | RelNo |  | Ex | no relevant discussion about how health related policy-making processes are affected by trade-related issues | Yes |
| Gonzalez, Through tobacco industry eyes: civil society and the FCTC process from Philip Morris and British American Tobacco’s perspectives (2012) |  |  |  |  |  | RelNo |  | Ex | Examines tobacco company's own analysis of the FCTC process and their subsequent tactics to influence tobacco control policies that fall outside the scope of this review | Yes |
| Gopinathan et al, Global governance and the broader determinants of health: A comparative case study of UNDP’s and WTO’s engagement with global health (2018) | Ananaly | MYes | DYes | Yes | Pmulti | RelYes | QualYes | Inc | Some nuggets of evidence on trade and health norms and involvement of WTO in global health governance | Yes |
| Gruning, Puffing Away? Explaining the Politics of Tobacco Control in Germany (2008) | AnPol |  |  |  |  | RelNo |  | Ex | Industry tactics but no discussion of use of trade and investment-related factors. May include later | Yes |
| Gruszczynski, COPing with the global tobacco epidemic: FCTC COP7 and its implications (2017) | AnPol | MNo | DNo | No | PTob | RelYes | QualCT | Inc | Provides mechanisms for economic overriding public health goals in international health agreement (FCTC) negotiations | Yes |
| Gruszczynski, The New Tobacco Products Directive and Wto Law: Much Ado About Nothing? (2012) | AnPol | MNo | DYes | No | PTob | RelYes | QualYes | Inc | Provides evidence for theory relating to misperceptions of risk for health polices in relation to WTO law | Yes |
| Gruszczynski, The Tbt Agreement and Tobacco Control Regulations (2013) | AnTIAr | MNo | DYes | No | PTob | RelYes | QualYes | Inc | Provides nuggets of evidence based on legal case analysis of policy space countries do have for tobacco regulation | Yes |
| Gruszczynski, The WTO and FCTC dispute settlement systems: Friends or foes? (2017) | Ananaly |  |  |  |  | RelNo |  | Inc | Evidence that trade objectives take precedent over public health even within international public health spaces | Yes |
| Gruszczynski, Tobacco and International Trade: Recent Activities of the FCTC Conference of the Parties (2016) | Ananaly | MNo | DYes | No | PTob | RelYes | QualYes | Inc | Provides one important nugget of evidence regarding how the regulatory environment is shaped to favour corporate interests | Yes |
| Hawkins et al, A Multi-Level, Multi-Jurisdictional Strategy : Transnational Tobacco Companies’ Attempts to Obstruct Tobacco Packaging Restrictions. (2019) | Ananaly | MYes | DYes | Yes | PTob | RelYes | QualYes | Inc | Provide evidence of industry venue shopping using different legal venues including international trade for a | Yes |
| Hawkins, A corporate veto on health policy? Global constitutionalism and Investor-State Dispute Settlement. (2016) | Case study | Myes | Dyes | Yes | Ptob | RelYes | QualYes | Inc | Provides nugget of evidence relating to tobacco industry use of trade and investment agreement legal threats | Yes |
| Hawkins et al, Alcohol industry influence on UK alcohol policy: a new research agenda for public health (2012) | AnCom | Mno | Dyes | No | Palc | RelYes | QualCT | Ex | Excluded since this was a commentary | Yes |
| Hawkins et al, Reassessing policy paradigms: a comparison of the global tobacco and alcohol industries (2018) | Ananaly | Myes | Dyes | Yes | Pmulti | RelYes | QualYes | Inc | How TNCs use trade liberalisation to consolidate power to then influence health policy processes | Yes |
| Hernandez-Aguado and Chilet-Rosell, Pathways of undue influence in health policymaking: a main actor's perspective (2018) | AnQual |  |  |  |  | RelNo |  | Ex | Excluded as it is not clear the article is concerned with TNCs or local private/corporate actors (whose activities in Spain would not necessarily be the result of trade and investment liberalization) | Yes |
| Higashi, The development of Tobacco Harm Prevention Law in Vietnam: stakeholder tensions over tobacco control legislation in a state-owned industry (2011) | AnPol |  |  |  |  | RelNo |  | Ex | Does not include discussions of international trade-related issues/arguments | Yes |
| Hirono et al, Negotiating healthy trade in Australia: Health impact assessment of the proposed Trans-Pacific Partnership Agreement (2015) | AnTIAp | MYes | DYes | No | Pmulti | RelYes | QualYes | Inc | Prospective analysis of potential health impacts of TPPA with some explanation of how the various provisions would affect health policy | Yes |
| Hirono et al, To what extent does a tobacco carve-out protect public health in the Trans-Pacific Partnership Agreement? (2014) | AnTIAp | MNo | DYes | No | PTob | RelYes | QualYes | Inc | Describes limitations of tobacco carve-out | Yes |
| Hirono, Is health impact assessment useful in the context of trade negotiations? A case study of the Trans Pacific Partnership Agreement (2015) | AnTIAp | Mno | Dyes | Yes | Pmulti | RelYes | QualYes | Ex | Excluded because more detailed results from the same HIA were included in "Negotiating healthy trade in Australia: Health impact assessment of theproposed Trans-Pacific Partnership Agreement (2015)" which has been included in the analysis | Yes |
| Holden et al, Cleavages and co-operation in the UK alcohol industry: a qualitative study (2012 | AnQual |  |  |  |  | RelNo |  | Ex | No relevant nuggets of evidence relating to trade | Yes |
| Holden et al, Trade Policy, Health, and Corporate Inﬂuence: British American Tobacco and China's Accession to the World Trade Organization (2010) | AnPol | MYes | DYes | Yes | PTob | RelYes | QualYes | Inc | Explores how BAT used China's accession to the WTO as a means to influence China's opening of their tobacco market | Yes |
| Jarman, Attack on Australia: Tobacco industry challenges to plain packaging (2013) | AnArb |  |  |  |  | RelNo |  | Ex | Legal analysis with no analysis of impact on policy processes | Yes |
| Jarman, Normalizing Tobacco? The Politics of Trade, Investment, and Tobacco Control (2019) | AnArb |  |  |  |  | RelNo |  | Ex | No sufficiently relevant nuggets of evidence for how health policy is influenced by trade and investment-related factors | Yes |
| Jarman, Trade policy governance: What health policymakers and advocates need to know (2017) | Ananaly | Mno | Dyes | No | Pmulti | RelYes | QualYes | Inc | Useful nugget of evidence relating to trade policy governance and potential strategies for health advocates to increase attention to health issues on the trade policy agenda | Yes |
| Jarman, When trade law meets public health evidence: the World Trade Organization and clove cigarettes (2012) | AnArb | MNo | DYes | No | PTob | RelYes | QualYes | Inc | Few nuggets of evidence for WTO substantive issues and health policy space. | Yes |
| Jenkin et al, Nutrition policy in whose interests? A New Zealand case study (2012) | AnQual | MYes | DYes | Yes | PNutr | RelYes | QualYes | Inc | Contributes to theories about perceptions and preferences and ideology | Yes |
| Jiang, Alliance between tobacco and alcohol industries to shape public policy (2013) | Ananaly |  |  |  |  | RelNo |  | Ex | Does not include discussion of trade or investment related issues so cannot transfer contextual factors that may have helped or inhibited health policy development. | Yes |
| Kasapila et al, Harmonisation of food labelling regulations in Southeast Asia: benefits, challenges and implications (2011) | AnPol |  |  |  |  | RelNo |  | Ex | Does not include analysis of trade-related issues/arguments | Yes |
| Kelsey, New-generation free trade agreements threaten progressive tobacco and alcohol policies (2012) | AnCom | Mno | DNo | No | Pmulti | RelYes | QualCT | Ex | Editorial without sufficient referencing | Yes |
| Kelsey, Regulatory Chill: Learnings from New Zealand's Plain Packaging Tobacco Law (2017) | AnPol | Mno | Dyes | Yes | Ptob | RelYes | QualYes | Inc | Includes analysis of how perceived risk from litigation, arguments from industry and bias towards minimal regulation can have a chilling effect on plain packaging regulations. Additionally, identifies that public support, international agreements and precedents from other countries can mitigate against indefinite chilling effect | Yes |
| Kelsey, The Trans-Pacific Partnership agreement: a gold-plated gift to the global tobacco industry? (2013) | AnTIAp | MNo | DYes | No | PTob | RelYes | QualYes | Inc | Novel nuggets of evidence about how the TPPA would operate with characteristics of a 'complex system' in that 'the whole is greater than the sum of its parts' and various chapters of the TPPA would potentially interact to have an effect on tobacco control policy development | Yes |
| Khan et al, The Transatlantic Trade and Investment Partnership: international trade law, health systems and public health (2014) | AnTIAp | MNo | DYes | No | Pmulti | RelYes | QualYes | Inc | Discusses issues of health actor participation in trade policy development processes | Yes |
| Knai et al. Systems Thinking as a Framework for Analysing Commercial Determinants of Health (2018) | Ananaly | Myes | Dyes | No | Pmulti | RelYes | QualYes | Ex | Excluded because relevant pieces of information were referenced from other empirical work | Yes |
| Koivusalo, Policy space for health and trade and investment agreements (2014) | AnPol |  |  |  |  | RelNo |  | Ex | Focused on generic provisions within TIAs and health services policies. Not sufficiently relevant to include in synthesis | Yes |
| Labonté et al, Framing international trade and chronic disease (2011) | AnFram | MNo | DYes | No | Pmulti | RelYes | QualYes | Inc | Provides some useful data on TNC expansion into developing markets and issues of policy space for reducing chronic diseases | Yes |
| Labonté et al, The Trans-Pacific Partnership Agreement and health: Few gains, some losses, many risks (2016) | AnTIAp | MNo | DYes | No | Pmulti | RelYes | QualYes | Inc | Provides evidence for theories relating to policy space in regards to new and updated provisions included in modern TIAs not previously found in WTO agreements | Yes |
| Labonté et al, The Trans-Pacific Partnership: Is it everything we feared for health? (2016) | AnTIAp | MYes | DYes | No | Pmulti | RelYes | QualYes | Inc | Evidence of how TTPA may impact health policy space | Yes |
| Labonté et al, Trade, investment and the global economy: Are we entering a new era for health? (2017) | AnTIAp | MNo | DYes | No | Pmulti | RelYes | QualYes | Inc | Provides nuggets of evidence relating to policy space | Yes |
| Lang, Rethinking the "harmonisation" of international trade and public health (2016) |  |  |  |  |  |  |  | FTNA |  | No |
| Lee et al, A "win-win situation overall": lessons from tobacco industry influence of the negotiation of the North American Free Trade Agreement (2018) | AnQual | Myes | Dyes | Yes | Ptob | RelYes | QualYes | Inc | Shows how tobacco industry influenced NAFTA negotiations | Yes |
| Lee et al, Bridging the divide: global governance of trade and health (2009) | Ananaly | Mno | Dyes | No | Pmulti | RelYes | QualYes | Inc | provided relevant nuggets on how WTO agreements are formed with limited consideration of health, thus creating the context in which health policies must be made. | Yes |
| Lee et al, Gaining access to Vietnam's cigarette market: British American Tobacco's strategy to enter ‘a huge market which will become enormous’ (2008) | AnQual | Myes | Dyes | Yes | Ptob | RelYes | QualYes | Inc | Relevant nuggets related to health policy as bargaining chips | Yes |
| Lee, What hinders implementation of the WHO FCTC Article 5.3? – The case of South Korea (2015) | AnPol |  |  |  |  |  |  | Ex | Focused on analysis of FCTC implementation, thus falls outside the scope of this review. | Yes |
| Lencucha and Thow, How Neoliberalism Is Shaping the Supply of Unhealthy Commodities and What This Means for NCD Prevention (2019) | Ananaly | MNo | DYes | No | Pmulti | RelYes | QualYes | Inc | Useful nuggets of evidence relating to the influence of ideology on NCD policy space | Yes |
| Lencucha et al, Navigating institutional complexity in the health sector: lessons from tobacco control in Kenya (2018) | AnPol |  |  |  |  | RelNo |  | Ex | Does not include discussion of trade or investment related issues so cannot transfer contextual factors that may have helped or inhibited health policy development | Yes |
| Lencucha et al, Global tobacco control and economic norms: an analysis of normative commitments in Kenya, Malawi and Zambia (2018) | AnPol | MYes | DYes | Yes | PTob | RelYes | QualYes | Inc | introduces new theory relating to international norms and internalisation of these norms in policy making | Yes |
| Lencucha et al, Rhetoric and the law, or the law of rhetoric: how countries oppose novel tobacco control measures at the World Trade Organization (2016) | AnArb | MYes | DYes | Yes | PTob | RelYes | QualYes | Inc | Qual study of arguments raised at WTO meetings by countries opposing tobacco regulations, these reflect the arguments repeatedly used by the tobacco industry | Yes |
| Leon and Ken, Food Fraud and the Partnership for a 'Healthier' America: A Case Study in State-Corporate Crime (2017) |  |  |  |  |  | RelNo |  | Ex | Industry tactics but no discussion of use of trade and investment-related factors. | Yes |
| Lester, Domestic Tobacco Regulation and International Law: The Interaction of Trade Agreements and the Framework Convention on Tobacco Control (2015) | AnLegal | MNo | DYes | No | PTob | RelYes | QualYes | Inc | Evidence of how the FCTC can be used in trade disputes to defend tobacco control regulations | Yes |
| Lie et l, The devil is in the detail: tobacco industry political inﬂuence in the Dutch implementation of the 2001 EU Tobacco Products Directive, (2015) | AnPol |  |  |  |  | RelNo |  | Ex | Discussed industry tactics used to influence tobacco control policy but no discussion of trade-related factors. | Yes |
| Lin, Exploring a modest balance for trade in tobacco, anti-tobacco smuggling and health concerns in light of the Dominican Republic-Cigarettes case(2008) | AnArb | MNo | DNo | No | PTob | RelYes | QualYes | Exc | Provides example of a case of restricted policy space for policy intended to reduce tobacco smuggling, however this policy area falls outside the scope of this review | Yes |
| Lo, et al, Reducing tobacco growing in Taiwan and government intervention: Challenges and opportunities (2010) | AnHist |  |  |  |  | RelNo |  | Ex | Discusses challenges of transitioning tobacco farmers to alternative crops, but issues mentioned are primarily technical or organisational, not trade-related factors. | Yes |
| Lyness, The alcohol industry, charities and policy influence in the UK (2014) | Andescript |  |  |  |  | RelNo |  | Ex | Excluded primarily on the basis that the study does not directly provide evidence of how TNCs influence health policy, thus evidence was considered not relevant enough | Yes |
| Mac-kenzie et al, The tobacco industry's challenges to standardised packaging: A comparative analysis of issue framing in public relations campaigns in four countries (2018) | AnQual | MYes | DYes | Yes | PTob | RelYes | QualYes | Inc | Provides nuggets of evidence of industry framing of plain packaging including use of trade-related legal framing | Yes |
| Mackenzie and Collin, 'A preferred consultant and partner to the Royal Government, NGOs, and the community': British American Tobacco's access to policymakers in Cambodia (2017) |  |  |  |  |  | RelNo |  | Ex | Industry uses economic issue framing but not explicitly in relation to international trade | Yes |
| MacKenzie and Collin, “Trade policy, not morals or health policy”: the US Trade Representative, tobacco companies and market liberalization in Thailand (2012) | AnQual | MYes | DYes | Yes | PTob | RelYes | QualYes | Inc | Discusses tobacco industry involvement in trade policymaking and negotiations | Yes |
| MacKenzie et al, To ‘enable our legal product to compete effectively with the transit market’: British American Tobacco’s strategies in Thailand following the 1990 GATT dispute (2016) | AnQual |  |  |  |  | RelNo |  | Ex | Focuses on analysis of legal and illegal trade in tobacco products in Thailand after liberalizing its tobacco market. Particularly focuses on how British American Tobacco's global strategy included illegal tobacco trade. Illegal tobacco trade falls outside the scope of this review, this review was therefore excluded | Yes |
| Mamudu et al, International trade versus public health during the FCTC negotiations, 1999-2003 (2011) | AnQual | MYes | DYes | Yes | PTob | RelYes | QualYes | Inc | Provides evidence of how global health norms developed at the international level are affected by trade objectives | Yes |
| Matanje Mwagomba et al, Alcohol policies in Malawi: inclusion of WHO “best buy” interventions and use of multi-sectoral action (2018) | AnPol |  |  |  |  | RelNo |  | Ex | Does not include discussion of trade-related issues | Yes |
| McCambridge et al, Alcohol industry involvement in policymaking: a systematic review (2018) | AnRev | MYes | DYes | No | PAlc | RelYes | QualYes | Inc | alcohol industry tactics including issue framing and relationship building | Yes |
| McCambridge et al, The challenge corporate lobbying poses to reducing society’s alcohol problems: insights from UK evidence on minimum unit pricing (2013) |  |  |  |  |  | RelNo |  | Ex | No sufficiently relevant nuggets of evidence relating to trade policy | Yes |
| McCambridge, Alcohol industry corporate social responsibility initiatives and harmful drinking: a systematic review (2018) |  |  |  |  |  | RelNo |  | Ex | Industry tactics but no discussion of use of trade and investment-related factors. | Yes |
| McNeill et al., Trade and Investment Agreements: Implications for Health Protection (2017) | AnRev | MNo | DYes | No | Pmulti | RelYes | QualYes | Inc | Provides nuggets of evidence on how trade rules may restrict health policy space | Yes |
| Mercille, Neoliberalism and the alcohol industry in Ireland (2016) | Ananaly |  |  |  |  | RelNo |  | Ex | No sufficiently relevant nuggets of evidence linking neoliberalism to trade and health policy | Yes |
| Mialon et al, Analysis of the corporate political activity of major food industry actors in Fiji (2016) | AnQual | MYes | DYes | Yes | PNutr | RelYes | QualYes | Inc | provides some counter evidence to policy chill theory but also supportive evidence for perceptions/preferences theory | Yes |
| Mitchell and Casben, Trade law and alcohol regulation: what role for a global Alcohol Marketing Code? (2016) | AnRev | MNo | DYes | No | PAlc | RelYes | QualYes | Inc | Provides some nuggets for theory on misperceptions about restrictiveness of international trade law and potential impact of international code on alcohol marketing | Yes |
| Mitchell and Sheargold, Protecting the autonomy of states to enact tobacco control measures under trade and investment  agreements (2014) | AnTIAp |  |  |  |  | RelNo |  | Ex | No sufficient new nuggets of evidence. Surverys measures to protect tobacco control policy space in TIAs but does not discuss in any detail the mechanisms by which TIAs restrict policy space | Yes |
| Mitchell et al, Public Health and the Trans-Pacific Partnership Agreement (2015) | AnTIAr | MNo | DYes | No | Pmulti | RelYes | QualYes | Inc | Provided nuggets of evidence relating to TPPA provisions and impact on health policy space | Yes |
| Mitchell, Australia’s Move to the Plain Packaging of Cigarettes and its WTO Compatibility (2010) | Ananaly | MNo | DYes | No | PTob | RelYes | QualYes | Inc | Includes analysis of how trade rules can be used to prevent regulatory development | Yes |
| Munro, Why the Distilled Spirits Industry Council of Australia is not a credible partner for the Australian government in making alcohol policy (2012) | Andescript |  |  |  |  | RelNo |  | Ex | Does not include analysis of trade-related issues/arguments | Yes |
| Nakkash, The passage of tobacco control law 174 in Lebanon: reflections on the problem, policies and politics (2018) | AnPol |  |  |  |  | RelNo |  | Ex | Does not include discussion of trade or investment related issues so cannot transfer contextual factors that may have helped or inhibited health policy development | Yes |
| O'Brien and Mitchell, On the Bottle: Health Information, Alcohol Labelling and the WTO Technical Barriers to Trade Agreement (2018) | AnWTOmt | MYes | DYes | No | PAlc | RelYes | QualYes | Inc | Relevant nuggets related to trade agreements and health policy space | Yes |
| O'brien et al, Marginalising health information: Implications of the Trans-Paciﬁc Partnership Agreement for alcohol labelling (2017) | AnTIAp | MNo | DYes | No | PAlc | RelYes | QualYes | Inc | Provides data on potential policy space restrictions placed on alcohol labelling by the TPPA (or TIAs modelling on the TTPA). Indicates unfounded concerns may result in regulatory chill | Yes |
| O'Brien, Australia’s double standard on Thailand’s alcohol warning labels (2018) | AnArb | MYes | DYes | Yes | PAlc | RelYes | QualYes | Inc | Analysis of WTO TBT Committee meeting minutes and evidence of industry influence in government positions | Yes |
| O'Brien, Commentary on 'Communicating Messages About Drinking': Using the 'Big Legal Guns' to Block Alcohol Health Warning Labels (2018) | AnCom | MNo | DYes | No | PAlc | RelYes | QualYes | Inc | Was a commentary but did provide relatively detailed analysis of trade challenges relating to alcohol labelling and potential policy space issues in TTPA and RCEP | Yes |
| O'Neill, Big Food without big diets? Food regimes and Kenyan diets (2015) | AnMix |  |  |  |  | RelNo |  | Ex | No relevant evidence | Yes |
| Oladepo et al., Analysis of tobacco control policies in Nigeria: historical development and application of multi-sectoral action (2018) | AnPol | MYes | DYes | Yes | PTob | RelYes | QualYes | Inc | provides evidence that agreement between gov and tobacco company to increase investment in local tobacco industry to boost exports may 'chill' gov domestic tobacco control policies | Yes |
| Orbinski et al, An Analysis of the Regional Convergence Process for the United Nations High-Level Meeting on Non-communicable Diseases: Lessons Learned. (2011) | Ananaly | Myes | Dyes | No | Pmulti | RelYes | QualYes | Inc | Provides nugget of evidence relating to trade taking precedent over health in global health instruments/declarations/guidelines etc | Yes |
| Pérez-Ferrer et al, Learning from international policies on trans fatty acids to reduce cardiovascular disease in low- and middle-income countries, using Mexico as a case study (2009) | AnPol |  |  |  |  | RelNo |  | Ex | No relevant discussion about how health related policy-making processes are affected | Yes |
| Petersmann, The Future of the Wto: From Authoritarian "Mercantilism" to Multilevel Governance for the Benefit of Citizens? (2010) | Ananaly |  |  |  |  | RelNo |  | Ex | No nuggets of evidence sufficiently relevant | Yes |
| Petersmann, How to Reconcile Health Law and Economic Law with Human Rights? Administration of Justice in Tobacco Control Disputes (2015) | AnTIAr |  |  |  |  | RelNo |  | Ex | No sufficiently relevant nuggets of evidence for how health policy is influenced by trade and investment-related factors | Yes |
| Public Citizen, Only One of 44 Attempts to Use the GATT Article XX/GATS Article XIV “General Exception” Has Ever Succeeded: Replicating the WTO Exception Construct Will Not Provide for an Effective TPP General Exception (2015) | AnArb | MNo | DYes | No | Pmulti | RelYes | QualYes | Inc | Analysis of the GATT general exception and the uncertainty that it will protect health policy space | Yes |
| Ranald, The Trans-Pacific Partnership Agreement: Reaching behind the border, challenging democracy (2015) |  |  |  |  |  |  |  | FTNA | Full text not available | No |
| Ravaro, Tobacco control policymaking in Portugal: vested interests or public health? (2015) | AnCom |  |  |  |  | RelNo | QualCT | Ex | This is a letter/commentary and does not provide sufficient relevant nuggets of evidence relating to how tobacco policy is affected by trade and investment-related factors. | Yes |
| Rimpeekool, Food and nutrition labelling in Thailand: a long march from subsistence producers to international traders (2015) | AnPol | MYes | DYes | No | PNutr | RelYes | QualYes | Inc | Some nuggets of evidence of WTO disputes within TBT committee restricting policy space for nutritional labelling in Thailand. | Yes |
| Royal College of Physicians, Fifty years since Smoking and health: progress, lessons and priorities for a smoke-free UK (2012) | AnPol | MNo | DYes | No | PTob | RelYes | QualYes | Ex | Evidence was taken from other empirical studies otherwise included in this review. This paper was therefore excluded as it did not provide any additional nuggets of evidence | Yes |
| Schram, When Evidence Isn't Enough: Ideological, Institutional, and Interest-Based Constraints on Achieving Trade and Health Policy Coherence (2018) | AnPol | MNo | DYes | No | Pmulti | RelNo | QualYes | Inc | Relevant nuggets of evidence of how ideology, institutions and interests within the trade and investment policy space may constrain policy recommendations made in the World Health Organization's Global Action Plan (GAP) on NCDs. | Yes |
| Scott et al, Food and beverage product reformulation as a corporate political strategy (2017) |  |  |  |  |  | RelNo |  | Ex | Industry tactics but no discussion of use of trade and investment-related factors. | Yes |
| Shankar et al, Policies for healthy and sustainable edible oil consumption: a stakeholder analysis for Thailand (2016) | AnPol |  |  |  |  | RelNo |  | Ex | Does not include discussion of trade or investment related issues so cannot transfer contextual factors that may have helped or inhibited health policy development | Yes |
| Shlomo-Agon, S. Clearing the Smoke: The Legitimation of Judicial Power at the WTO (2015) |  |  |  |  |  | RelNo |  | Ex | No nuggets of evidence sufficiently relevant to the research topic | Yes |
| Smith et al, ‘‘Working the System’’—British American Tobacco’s Influence on the European Union Treaty and Its Implications for Policy: An Analysis of Internal Tobacco Industry Documents (2010) | AnPol | MYes | DYes | Yes | PTob | RelYes | QualYes | Inc | Supports theory that TNCs attempt to influence policy process through international agreements and seek to force a shift away from use of the precautionary principle in regulatory decision-making | Yes |
| Smith et al, Corporate Coalitions and Policy Making in the European Union: How and Why British American Tobacco Promoted "Better Regulation" (2015) | AnMix | MYes | DYes | Yes | PTob | RelYes | QualYes | Ex | Does not explicitly discuss how trade rules or norms were used by TNCs to influence tobacco policy. | Yes |
| Smith et al, The atlas network: a "strategic ally" of the tobacco industry (2017) |  |  |  |  |  | RelNo |  | Ex | Industry tactics but no discussion of use of trade and investment-related factors. | Yes |
| Snowdon and Thow, Trade policy and obesity prevention: challenges and innovation in the Paciﬁc Islands (2013) | Ananaly | MNo | DYes | No | PNutr | RelYes | QualYes | Inc | Provides one useful nuggets of evidence regarding nutrition policy space | Yes |
| Studlar, Punching above their weight through policy learning : tobacco control policies in Ireland (2015) | AnPol |  |  |  |  | RelNo |  | Ex | Does not include discussion of trade or investment related issues so cannot transfer contextual factors that may have helped or inhibited health policy development | Yes |
| Stumberg, Safeguards for Tobacco Control: Options for the TPPA (2013) | AnTIAp | MNo | DYes | No | PTob | RelYes | QualYes | Inc | Very detailed legal analysis of how WTO GATS/GATT may restrict policy space and generate regulatory chill despite the health exception | Yes |
| Swinnen, The Political Economy of Agricultural and Food Policies: Recent Contributions, New Insights, and Areas for Further Research (2010) |  |  |  |  |  | RelNo |  | Ex | No relevant discussion about how health related policy-making processes are affected | Yes |
| Sy, TPPA and tobacco control: threats to APEC countries (2014) | AnTIAp | MYes | DYes | No | PTob | RelYes | QualYes | Inc | Evidence of how TTPA may impact tobacco control policy space | Yes |
| Thaiprayoon and Smith, Capacity building for global health diplomacy: Thailand's experience of trade and health (2015) | AnQual | Myes | Dyes | Yes | Pmulti | RelYes | QualYes | Inc | Provides evidence of a nutrition policy with implications for trade but developed in such a way as to comply with 'non-discriminatory' and 'national treatment' trade rules. |  |
| Thow et al, Development, implementation and outcome of standards to restrict fatty meat in the food supply and prevent NCDs: learning from an innovative trade/food policy in Ghana (2014) | AnPol | MYes | DYes | Yes | PNutr | RelYes | QualYes | Inc | Provides evidence of a nutrition policy with implications for trade but developed in such a way as to comply with 'non-discriminatory' and 'national treatment' trade rules. | Yes |
| Thow et al, Nutrition labelling is a trade policy issue: lessons from an analysis of specific trade concerns at the World Trade Organization (2017) | AnWTOmt | MYes | DYes | No | PNutr | RelYes | QualYes | Inc | Relevant nuggets related to trade agreements and health policy space | Yes |
| Thow et al, Trade and food policy: Case studies from three Paciﬁc Island countries (2010) | AnPol | MYes | DYes | Yes | PNutr | RelYes | QualYes | Inc | analysis of policy process and contextual factors that supported adoption of potentially trade-restrictive policies | Yes |
| Thow et al, Will the next generation of preferential trade and investment agreements undermine prevention of noncommunicable diseases? A prospective policy analysis of the Trans Pacific Partnership Agreement (2015) | AnTIAp | MNo | DYes | No | PNutr | RelYes | QualYes | Inc | Provides a prospective analysis of potential impacts the TPPA may have on nutrition policy space | Yes |
| Thow et al. Improving policy coherence for food security and nutrition in South Africa: a qualitative policy analysis (2018) | AnQual | MYes | DYes | Yes | PNutr | RelYes | QualYes | Inc | Provides some evidence in support of and other evidence indicating a need for refinement/change of theories relating to ideology and perceptions. | Yes |
| Thow, Food supply, nutrition and trade policy: reversal of an import ban on turkey tails (2017) | Case study | MNo | DYes | No | PNutr | RelYes | QualYes | Inc | Provides nugget of evidence of policy chill | Yes |
| Ulucanlar et al, Representation and Misrepresentation of Scientific Evidence in Contemporary Tobacco Regulation: A Review of Tobacco Industry Submissions to the UK Government Consultation on Standardised Packaging (2014) |  |  |  |  |  | RelNo |  | Ex | Industry tactics but no discussion of use of trade and investment-related factors. | Yes |
| Ulucanlar, The Policy Dystopia Model: An Interpretive Analysis of Tobacco Industry Political Activity (2016) | AnFram | Myes | Dyes | No | Ptob | RelYes | QualYes | Inc | Nugget of evidence on tobacco companies use of GATT and TBT disputes despite lack of legal basis | Yes |
| Van Harten, investment Treaties and the Internal Vetting of Regulatory Proposals: A Case Study from Canada (2016) | AnQual | Myes | Dyes | Yes | Pmulti | RelYes | QualYes | Inc | Exposes very close relationship between trade department and industry in Canada and that health policy makers may internalise trade obligations in their decision-making | Yes |
| Von Tigerstrom, How do international trade obligations affect policy options for obesity prevention? Lessons from recent developments in trade and tobacco control (2013) | AnMix | MNo | DYes | No | PNutr | RelYes | QualYes | Inc | Evidence of potential for trade rules to restrict policy space | Yes |
| Voon, Flexibilities in WTO law to support tobacco control regulation (2013) | AnTIAp | MNo | DYes | No | PTob | RelYes | QualYes | Inc | Provides expert opinion on the flexibilities for tobacco regulation available in WTO agreements relevant to explanatory theory about missed opportunities | Yes |
| Walls et al, International trade and investment: still the foundation for tackling nutrition related non-communicable diseases in the era of Trump? (2019) | Ananaly |  |  |  |  | RelNo |  | Ex | No relevant data | Yes |
| Waqa et al., Exploring the dynamics of food-related policymaking processes and evidence use in Fiji using systems thinking (2017) | AnQual |  |  |  |  | RelNo |  | Ex | No relevant discussion about how health related policy-making processes are affected | Yes |
| Weishaar, Best of enemies: Using social network analysis to explore a policy network in European smoke-free policy (2015) | AnPol |  |  |  |  | RelNo |  | Ex | No nuggets of evidence sufficiently relevant | Yes |
| Weishaar, Global health governance and the commercial sector: A documentary analysis of tobacco company strategies to influence the who framework convention on tobacco control, (2012) | AnQual | MYes | DYes | Yes | PTob | RelYes | QualYes | Inc | Provided nuggets of evidence on how tobacco companies attempt to shape perceptions and preferences at the global policy level (but these tactics have all also been used at the national level). Includes mention of threat of non-compliance with trade agreements | Yes |
| Weiss, Trading Health? UK Faculty of Health Policy Report on the Transatlantic Trade and Investment Partnership (2015) | AnTIAp | MNo | DYes | No | Pmulti | RelYes | QualYes | Inc | Includes nuggets of evidence relating to industry use of trade rules to argue against alcohol regulations | Yes |
| WHO, Confronting the tobacco epidemic in a new era of trade and investment liberalization (2012) | AnEnv | MNo | DYes | No | PTob | RelYes | QualYes | Inc | Relevant nuggets of evidence relating to Tobacco industry invocation of trade and investment agreements in attempts to resist regulation and also the utility and conflict between the FCTC and international trade agreements | Yes |
| WHO, Framework Convention on Tobacco Control. Economically sustainable alternatives to tobacco growing (in relation to Articles 17 and 18 of the WHO Framework Convention on Tobacco Control) (2018) | Ananaly | Mno | Dyes | No | Ptob | RelYes | QualYes | Inc | Evidence of some LMICs perceiving tobacco industry as important for economic growth | Yes |
| William and Shaarani, Harmonisation of food labelling regulations in Southeast Asia: benefits, challenges and implications (2011) |  |  |  |  |  | RelNo |  | Ex | No relevant data | Yes |
| Zeigler, The alcohol industry and trade agreements: a preliminary assessment (2008) | AnTIAr | MNo | DYes | No | PAlc | RelYes | QualYes | Inc | Alcohol industry tactics including attempts to shape trade rules | Yes |

**Codes used**

**Type of study/source**

Ananaly = other analysis; Anarb= analysis of a arbitral decision/dispute; Ancom= commentary/editorial/op ed; Anmix= mixed methods; Anfram= conceptual framework development; Andescript= descriptive; Anpol= policy analysis; AnTIAp= prospective analysis of a trade agreement; AnTIAr= retrospective analysis of a trade agreement; AnWTOmt= Analysis of WTO committee meetings; Anqual= qualitative analysis; Anrev= review; Anquant= quantitative analysis; Anlegal= legal analysis; case study= case study

**Methods stated**

Myes= methods stated; Mno= methods not stated

**Data source stated**

Dyes= data source stated or referenced; Dno= data sources and/or references not provided

**Policy area**

Palc= alcohol; Ptob= tobacco; Pnutr= nutritin; Pmulti= multipe policy areas

**Inclusion criteria: Relevant**

RelYes= yes; RelNo=no

**Inclusion criteria: reliable**

QualYes= yes; QualNo= no; QualCT= can’t tell

**Decision to include or exclude:**

Inc= include; Ex= exclude
