# Supplementary material for: Corporate power and the international trade regime preventing progressive policy action on non-communicable diseases: a realist review
Source: Health Policy Plan. 2020 Dec 4;36(4):493–508. doi: 10.1093/heapol/czaa148 (PMC8128013; doi:10.1093/heapol/czaa148)
Supplement: czaa148_Supp [file czaa148_supp.zip › Supplementary Text II_ScreeningTool.docx]

**Supplementary Text II:**

**Screening Tool**

| **Author, title of publication (year):** | **Type of study/source** | **Method stated** | **Data source stated (or referenced)** | **Empirical research** | **Policy area** | **Inclusion/Exclusion criteria: Relevant** | **Inclusion/Exclusion criteria: Reliable** | **Decision to include/exclude?** | **Reason for decision** | **Full text accessible** |
| --- | --- | --- | --- | --- | --- | --- | --- | --- | --- | --- |
|  |  |  |  |  |  |  |  |  |  |  |
|  |  |  |  |  |  |  |  |  |  |  |
|  |  |  |  |  |  |  |  |  |  |  |
