# Supplementary material for: Corporate power and the international trade regime preventing progressive policy action on non-communicable diseases: a realist review
Source: Health Policy Plan. 2020 Dec 4;36(4):493–508. doi: 10.1093/heapol/czaa148 (PMC8128013; doi:10.1093/heapol/czaa148)
Supplement: czaa148_Supp [file czaa148_supp.zip › SuppTextIII_Trade rules.docx]

**Supplementary Text III:**

**International trade rules with the potential to restrict regulation of risk commodities**

Since the break-down of the World Trade Organisation’s (WTO) Doha Round negotiations, developed countries with support from TRCCs, have pursued progressively expansive trade and investment liberalization through alternative means than those provided by the multilateral WTO process (Gleeson and Friel, 2013, Friel et al., 2013, Walls and Smith, 2015). These include bilateral investment treaties (BITs) and, increasingly, large regional trade and investment agreements (RTAs) (Friel et al., 2013, Gleeson and Friel, 2013, Walls et al., 2015, Friel et al., 2015). Compared to earlier agreements, contain ‘WTO-plus’ provisions that are deeper than minimum WTO obligations (Baldwin, 2011, WTO, 2013, Bacchetta et al., 2011) and ‘WTO-extra’ provisions that extend ‘behind borders’ to reduce what are considered to be non-tariff barriers to trade (Baldwin, 2011). RTAs including the Trans-Pacific Partnership Agreement (TPPA) which, despite US withdrawal, is set to be concluded between 11 Asia-Pacific Rim countries (Gleeson and Friel, 2013, Kelsey, 2012), the Regional Comprehensive Economic Partnership (RCEP) between 16 Asia-Pacific countries (Townsend et al., 2016) and the Comprehensive Economic and Trade Agreement (CETA) between Canada and the EU (EU, 2016), are likely to be particularly important given the collective size of the economies involved, the political power of many of the negotiating governments (Hawkins and Holden, 2016), and the political leverage held by large TRCCs originating from/operating in negotiating countries. However, currently the majority of developing countries obligations remain limited to WTO rules, with governments cautious not to make deeper obligations that may negatively impact social and economic development. So, while more recent prospective analyses argue that RTAs may have particularly restrictive effects on health policy processes, we have included an analysis of relevant WTO agreements as well as WTO-plus and WTO-extra provisions included within more recently negotiated RTAs. While we aim to cover all key trade-related provisions in this analysis, investment protection provisions included in many RTAs and BITs are covered in a separate but related work.

**General Agreement on Tariffs and Trade (GATT)**

GATT lays out rules for treatment of products in international trade. The most important of these are the obligations of non-discrimination: the ‘most-favoured nation’ (MFN) in Article I requires that states give equally favourable treatment to ‘like’ products of all other members; the ‘national treatment’ in Article III requires that states treat foreign products at least as favourably as ‘like’ domestic products when they regulate (von Tigerstrom, 2013). This constrains governments’ ability to use measures that discourage consumers from developing a taste from imported alcohol products, particularly those with higher alcohol content or those targeted at adolescents (Zeigler, 2009). There have been a number of such cases, for example the US, Canada and the European Commission have invoked the national treatment rule over Japan’s high taxes on import alcohol successfully arguing that vodka, gin, rum and whiskey and other spirits are ‘like’ Japan’s traditional spirit shochu, as a result, liquor prices in Japan dropped (Baker et al., 2014), a similar ruling was made against Korea (Mamudu et al., 2011), the UK, Ireland and Nordic countries (Zeigler, 2009). In a Chilean case a WTO panel rules that spirits with higher alcohol content could not be taxed at a higher rate since this in effect favoured Chilean liquor pisco that had lower percentage alcohol than imported spirits (Mamudu et al., 2011). The US has also used GATT rules to argue that Canadian provincial liquor taxation levels and minimum pricing were discriminatory against less expensive imported US beer (Mamudu et al., 2011).

GATT articles XIV and XX recognize the protection of human health as an interpretive principle giving countries the space to adopt trade restrictive measures when it is is ‘necessary to protect human, animal or plant life and health’ (Labonté et al., 2011). However, as discussed in detail below, a country must satisfy three successive threshold tests. Firstly, they must prove that the policy in question is designed to protect human, animal or plant life or health; contributes to a legitimate health objective and is necessary to protect health with no alternative less trade restrictive measure available; and is not arbitrarily or unjustifiably discriminatory between countries and is not a disguised restriction on trade (Citizen, 2015). Some argue this provision has been interpreted narrowly (Baker et al., 2014), a position potentially supported by a 2015 report finding that just one of 44 attempts to invoke Article XX, (or the equivalent provision in GATS), had ever been successful. The exception was considered relevant in just 33 of these cases, and in most of these (18 cases) the governments involved were unable to sufficiently prove the measures were ‘necessary to’ or ‘related to’ protecting health or conserving natural resources (Citizen, 2015). After lobbying by the US Cigarette Exporters’ Association, the US challenged Thailand under GATT for prohibiting imported cigarettes (MacKenzie and Collin, 2012, Lester, 2015). Thailand attempted to use the exemption for public health protection arguing that opening their market would increase smoking prevalence (MacKenzie and Collin, 2012). For example, the Thai government argued that increased competition ‘would lead to the use of better marketing techniques (including advertising), a wider availability of cigarettes, a possible reduction of their prices, and perhaps improvement in their quality’ (MacKenzie and Collin, 2012). Thailand also claimed ‘the United States cigarette industry would exert great efforts to force governments to accept terms and conditions which undermined public health and government were left with no effective tool to carry our public health policies’ (Lester, 2015). This argument required demonstrating with sufficient evidence that market restrictions were necessary to prevent increased smoking prevalence and was the least trade restrictive option available (MacKenzie and Collin, 2012). The panel rejected Thailand’s argument ruling that the restriction breeched non-discrimination obligations under GATT and suggested that ‘there were various measures consistent with the General Agreement which were reasonably available to Thailand to control the quality and quantity of cigarettes smoked and which, taken together, could achieve the health policy goals that the Thai government pursues by restricting the importation of cigarettes inconsistently with Article XI:I’ (MacKenzie and Collin, 2012). Arguably, this can be interpreted as a narrow interpretation of the health exception by WTO panels showing insensitivity to the challenges faced by developing countries in regulating tobacco when multi-national tobacco corporations enter then market (Lester, 2015). Others have gone further to suggest this case also indicates that ‘necessity’ can be interpreted ‘with a bias against rules that discriminate against foreign investors’ (Zeigler, 2009). The US used the same argument to force open Taiwan and South Korea’s market to foreign tobacco companies (Mamudu et al., 2011). In part due to the previous failed attempts to invoke Article XX but also due to the inclusion of investment chapters (discussed in a related review) providing investors extensive rights and privileges that can conflict with governments’ attempts to regulate in the interest of health or the environment, there is significant concern amongst some experts that importing Article XX into new multilateral agreements like the TPP would be ineffective at protecting health and the environment (Citizen, 2015).

Tariffs can be an important source of government revenue for LMICs and commitments under the WTO to lower tariffs before adequate alternative taxation mechanisms are developed has been found to reduced public revenue in many LMICs (Thow et al., 2015). At the same time the legal, regulatory and other infrastructure required to comply with trade agreements can place significant additional financial burden on LMICs (Thow et al., 2015). This may have indirect impacts on the public finances available for public health policy development and implementation (Baker et al., 2016).

More recently negotiated trade and investment agreements including the TTIP propose deeper reductions or elimination of tariffs on some certain risk commodities including processed foods and alcohol. Such reductions on processed foods are predicted to generate one of the largest percentage increases in imports of goods by sector (Khan et al., 2015). In the TTIP for example, evidence indicates imports into the EU of US agri-food produce may increase two-fold by 2025, although this is from a low baseline. The CETA agreement eliminates all import tariffs on alcohol and Spirits Canada have stated they expect to double their exports to Europe, particularly targeting Eastern Europe (Khan et al., 2015). Increased availability is likely to lead to reduced cost and increasing the availability of unhealthy foods and alcohol which may pose challenges to developing effective nutrition and alcohol policy (Khan et al., 2015).

It may however be possible for governments to counter such tariff reductions with tax, since it is not considered a technical barrier to trade. For example, while NAFTA resulted in a significant reduction in tariffs on soft drinks in Mexico, after pressure from public health stakeholders, the Mexican government introduced a tax on soft drinks (Khan et al., 2015). However, similar fiscal policies to reduce the affordability of other certain unhealthy foods may be challenging to apply without TRCCs attempting to raise a trade challenge since it is possible that a regulation or tax rate could affect an imported product or product from a specific country disproportionately as compared to an arguably ‘like’ domestic product (Thow et al., 2015, von Tigerstrom, 2013). For example, a policy of applying variable tax rates to different food products in an effort to inﬂuence consumption patterns, where those with lower rates are generally locally produced foods; or labelling regulations that applies only to specific food product categories, where the majority of those types of products are imported from a particular country (von Tigerstrom, 2013). Discrimination would only be allowed on public health grounds if the measure is supported by sufficient evidence that it is ‘necessary’ to achieve certain health objectives, it is not a disguised restriction on trade and no other reasonable less trade-restrictive alternative is available (von Tigerstrom, 2013).

**Agreement on Sanitary and Phytosanitary Measures (SPS)**

The WTO SPS agreement sets out procedural and substantive requirements aimed at preventing domestic health and safety standards from unnecessarily impeding international trade (directly or indirectly) (Labonte et al., 2016). This includes any measure adopted to protect consumers from food-borne risks (e.g. from food additives, contaminants, toxins or disease causing organisms) and protect consumers from disease-related risks including import bans, processing and product standards and information tools such as labelling requirements (Labonte et al., 2016). Measures are generally considered consistent with the SPS if they are based on a relevant international standard (e.g. Codex Alimentarius Commission) (Friel et al., 2019). It is a significant concern however that such standards are highly influenced by multination food corporations (Friel et al., 2019).

When a domestic measure is stricter than international standards, the state must scientifically justify the measure based on a risk assessment that may, according to the SPS, be ‘based on’ minority scientific opinion, essential for allowing application of the precautionary principle (Labonte et al., 2016, Labonté et al., 2017

). TRCCs however have supported a move in more recently negotiated agreements to higher levels of evidential requirements to justify new regulations, including in SPS regulations. For example, PMI’s comments on the TPP states that they support ‘negotiations that promote… science-based regulations’(Stumberg, 2013) and the TPP’s draft SPS Chapter reflects this, stating that if a country’s regulation exceeds international standards, they will be required to provide ‘documented and objective scientific evidence’ potentially raising the level of evidence required to defend an SPS regulation related to food, alcohol or tobacco that exceeds international standards, effectively curtailing the use of the precautionary principle (Labonte et al., 2016). Similarly, the draft SPS Chapter in the TTIP requires that Members’ SPS measures are based on international standards or scientific risk assessment with the right to apply the precautionary principle only to the extent necessary. This demand for higher levels of evidence to justify a trade restrictive health policy included in more recent trade agreements may contribute to a political reluctance to implement novel policies to address the complex problems of reducing consumption of unhealthy food and alcohol since the impact of such policies cannot be directly measured a priori but rather may often require a ‘learning by doing’ approach (Baker et al., 2017).

**Agreement on Trade-Related Aspects of Intellectual Property Rights (TRIPS)**

The TRIPS agreement deals with intellectual property rights including trademarks and patents making it relevant to alcohol, food and tobacco control policies. TRCCs have specifically called for a ‘TRIPS-plus’ chapter in the TPP which may protect their use of trade-marks allowing TRCCs to challenge the introduction of, plain packaging or potentially other forms of risk commodity advertising, which according to legal experts, is not possible under the existing WTO TRIPS Agreement that protects their right to register a trademark but not to use it (Fooks and Gilmore, 2014, Hirono et al., 2015, Mitchell et al., 2014). This has not prevented TRCCs arguing that existing TRIPS rules have been violated by regulations restricting their use. Most recently, PMI argued Australia’s plain packaging regulation violated TRIPS. Earlier in 1995 after Guatemala introduced legislation aligned with the WHO’s International Code on Marketing of Breastmilk Substitutes that prohibited marketing by showing pictures of babies, on behalf of Gerber Foods, the US initiated a WTO dispute threatening Guatemala with withdrawal of most favoured nation trading status for violating trademark rules (Shekhar, 2016, Labonté et al., 2011). This threat likely played a role in the Guatemalan Supreme Court ruling that Gerber would be exempt from an obligation to comply with the labelling regulations (Shekhar, 2016).

**Technical Barriers to Trade Agreement (TBT)**

The TBT agreement covers technical regulations and standards including many relevant to tobacco, alcohol and food policy including packaging, labelling or product content (e.g. sugar, salt or trans-fat content) regulations that have an effect on investors (von Tigerstrom, 2013). Two key TBT Agreement obligations that affect risk commodity regulatory space are the non-discrimination obligation and the requirement to use ‘least trade restrictive measures’ to achieve a health objective (Lester, 2015). Harmonization is also a key element of the TBT agreement and members are expected, where possible, to use international standards as the basis of technical regulations (Lester, 2015) and in this case are ‘presumed not to create an unnecessary obstacle to international trade’ (von Tigerstrom, 2013). Over the past five years, nutrition and alcohol labelling have been repeatedly raised in the TBT Committee as a specific trade concern. Members have argued such regulations may be more trade restrictive than necessary with other less trade restrictive alternatives available, are not based on international standards and in this context, insufficient scientific evidence, and/or were discriminatory (von Tigerstrom, 2013, Thow et al., 2017). For example eleven countries raised concerns that Peru’s proposed front of package interpretive nutrition labelling may be more trade restrictive than necessary to achieve the stated objective of reducing obesity to combat NCDs and was not based on sufficient scientific evidence to demonstrate this (Rimpeekool et al., 2015, Thow et al., 2017); concerns were also raised that Chile’s labelling of foods high in fat, sugar, calories or salt deviated from international standards, may not have a scientific basis and would be more trade restrictive than necessary (Thow et al., 2017); comments were made that Indonesia should consider less trade restrictive alternative measures to their proposed labelling (for example education campaigns); and some members complained Thailand’s proposed labelling discriminated against snack foods (Thow et al., 2017). Since 2010 there have also been nine alcohol health warning labels that have been raised as a specific trade concern (O’Brien and Mitchell, 2018) most commonly on the grounds that the proposal is more trade restrictive than necessary and/or that the measure is not consistent with international standards (O’Brien and Mitchell, 2018). In 2011 twenty WTO members complained that Canada’s ‘Cracking Down on Tobacco Marketing Aimed at Youth Act’ initiative that prohibited tobacco products containing certain additives would effectively exclude blended cigarettes using tobacco from specific countries including Malawi, Kenya and Uganda, which was in violation of the TBT (Collin, 2012, Gruszczynski). Brazil’s even stricter ban on the use of additives with the goal of reducing the attractiveness of smoking has also been subject to discussion at the TBT Committee (Gruszczynski). The comprehensive scope of the ban could in effect eliminate almost all blended cigarettes from the Brazilian market which account for nearly all cigarettes sold, as such the measure was criticised as being more trade restrictive than necessary and without sufficient scientific basis (Gruszczynski). Also under Article II of the TBT, members are obligated to notify the WTO of proposed technical regulations and allow sufficient time to receive and take into consideration other members’ comments (Thow et al., 2017, O’Brien and Mitchell, 2018).

The TBT chapter in the TPP goes beyond WTO TBT commitments in two main ways. First it provides new avenues for TRCCs to participate in regulatory development ‘on terms no less favourable that those it accords to its own persons’ creating the potential of regulatory capture (Labonté et al., 2017

). Secondly, while the WTO TBT infers that a regulation that complies with an existing international standard is not in violation of the agreement, the TPP TBT calls on members to co-operate in developing international standards such that they ‘do not create unnecessary barriers to international trade’. This has the potential to weaken international health standards and by default also domestic health regulations (Labonté et al., 2017

). Further, the TBT states that ‘nothing…shall prevent a Party from adopting or maintaining technical regulations or standards’ provided, however, that these are “in accordance with its rights and obligations under this Agreement’ (art.8.3), so only health regulations that are otherwise compliant with the TBT are permitted (Labonté et al., 2017

).

**General Agreement on Trade in Services (GATS)**

GATS commitments state the extent of access foreign service providers are allowed (Zeigler, 2009) and each country chooses which specific sectors they wish to open to international trade. GATS contains two main obligations that apply to all sectors covered under trade in services: non-discrimination (similar to the GATT and TBT agreements), and a transparency provision obligating prompt notification of all measures affecting trade in services (von Tigerstrom, 2013, Zeigler, 2009). Deeper commitments including restrictions on domestic regulation of services only apply when a country has specifically committed to such for a certain service sector or type of trade in services (von Tigerstrom, 2013). Services relevant to risk commodities that may be covered under GATS include for example packaging, retail and distribution and advertising (Stumberg, 2013). Relevant provisions include the market access provisions that prohibits limitations on the number of service suppliers, service operations or participation of foreign capital in sectors covered under the agreement (Zeigler, 2009). Many members have made commitments under ‘distribution services’ for example which may limit policy-makers’ capacity to restrict alcohol and tobacco supply by limiting retail outlets, total volume or sales (Zeigler, 2009).

More recently negotiated trade agreements contain GATS plus provisions. In the WTO rules on market access prohibitions of quotas apply to a ‘positive list’ of sectors (i.e. a select list of agreed sectors) in a country’s schedule of commitments (Stumberg, 2013). The TPP however is a ‘negative list’ meaning the rule applies to all sectors except those included in the list. It may well be more challenging for public health policy makers to get a sector on the negative list as opposed to keep it off a positive list. Consequently, the TPP could affect cross border distribution of risk commodity products under market access for most TTP countries (Stumberg, 2013). Other public health researchers are concerned GATS may impact countries seeking to implement restrictions on marketing of food and beverages, including to children as well as alcohol advertising as this may be considered a barrier to cross-border advertising (a type of trade in services) (Hirono et al., 2015). This would however only be the case if the country had made specific commitments to disciplines on domestic regulation in the advertising sector (von Tigerstrom, 2013) as may be the case in more recent TIAs including the TPP which may potentially add to GATS commitments on domestic regulation and expand coverage to additional sectors, e.g. the advertising sector (Stumberg, 2013).

**Regulatory coherence and transparency**

Regulatory coherence chapters have only been recently introduced into trade agreements. They introduce requirements on the degree of transparency in domestic policy development processes and institutionalize the right of private actors to participate (Friel et al., 2019, Gruszczynski, 2017). Requirements include providing public access to documentation relevant to all regulatory measures, potentially giving TRCCs access to more information they can use to litigate (Kelsey, 2013). Governments are also required to provide opportunities for ‘interested persons’ and other parties to have input in policy-making processes (Labonte et al., 2016). Further, both the TPP and TTIP proposed the establishment of an inter-governmental body comprised of regulators and industry for the purpose of driving regulatory harmonization between countries, providing a further avenue for TRCC influence (Weiss, 2015). KORUS (The United States–Korea Free Trade Agreement) for example, contains a provision in the TBT chapter, stating that ‘Each Party shall allow persons [a national or an enterprise] of the other Party to participate in the development of standards, technical regulations, and conformity assessment procedures’ (Thow et al., 2015). A similar provision exists in the leaked draft proposal for the regulatory coherence chapter of the TPP (Kelsey). These provisions allow for industry from other countries to participate in policy development with limited scope to restrict their input (Thow et al., 2015).

Regulatory coherence chapters also encourage the use of regulatory impact assessments (RIAs) which TRCCs, specifically British American Tobacco, have worked since the 1990s to have embedded in policy-making processes (Schram et al., 2018, Thow et al., 2015, Labonte et al., 2016). RIAs creates a structure for developing regulations that use an economic framework of analysis, ensures early corporate involvement in policy development and formalizes the ability of TRCCs to exploit the information asymmetries between government and corporations (Smith et al., Fooks and Gilmore, 2014). The tobacco industry has, for example, delayed implementation of graphic warning labels in the US by questioning the Food and Drug Administration’s (FDA) RIA, a similar strategy has been used in New Zealand (Sy and Stumberg, 2014). Industry efforts to enshrine RIA in policy-making processes has more recently been reflected in their recommendation for international trade agreements including for example the TPP (Fooks and Gilmore, 2014). In line with this recommendation a leaked draft of the TPP Regulatory Coherence Chapter proposed that governments should endeavour to conduct RIAs as ‘best practice’ on all regulations under development aiming for decisions to be ‘based on the best reasonably obtainable scientiﬁc, technical, economic and other information’ (Mitchell et al., 2014). Additionally, it recommends for national bodies to be established to promote coordination of policy development across all departments and with the authority to review compliance with ‘good regulatory practice’ (Stumberg, 2013, Friel et al., 2013, Labonte et al., 2016) presumed to include the completion of a ‘pro-market and pro-business’ RIA (Kelsey, 2013). This may generate barriers for innovative public health policies, complicate the policy process and increase the cost of public policy development.

It is important to note that many, particularly HICs countries already widely adopt pro-business assessment of policies under consideration and perceive industry consultation as necessary and valuable. For example the US, Australia and New Zealand already follow a ‘best practice’ approach to domestic policy/regulatory development based on consideration of a set of pro-market factors and following a pro-business process (Kelsey, 2013). Regulatory coherence and transparency chapters however would create significant new obligations for LMICs which may deter policy development or increase the risk of attracting threats of an investor-state dispute and lose of such a dispute were it to be pursued (Kelsey, 2013). Together regulatory coherence and transparency obligations are predicted to provide TRCCs with additional legitimate tools and fora to influence health policy-making processes (Fooks and Gilmore, 2014, Kelsey, 2013, Ruckert et al., 2017, Hirono et al., 2015).

**Harmonization**

Under the WTO’s TBT agreement governments are not necessarily required to adhere to agreed regulatory schemes between countries. Supported by TRCCs, however both the proposed TPP and TTIP included chapters on harmonization of technical norms and standards which seek to streamline and harmonize regulations across countries, limiting ‘regulatory diversity’. These provisions have the potential to restrict countries’ space to implement stricter than agreed standards on for example front of package food labelling (Thow et al., 2015).

**References**

BACCHETTA, M., BEVERELLI, C., HANCOCK, C., KECK, A., NAYYAR, G., NEE, C., PIERMARTINI, R., ROCHA, N., ROY, M., RUTA, M., TEH, R. & YANOVICH, A. 2011. World Trade Report 2011. World Trade Organisation.

BAKER, P., FRIEL, S., SCHRAM, A. & LABONTE, R. 2016. Trade and investment liberalization, food systems change and highly processed food consumption: a natural experiment contrasting the soft-drink markets of Peru and Bolivia. *Globalization and Health,* 12.

BAKER, P., GILL, S., FRIEL, S. & KAY, A. 2017. Generating political priority for regulatory interventions targeting obesity prevention: An Australian case study *Social Science and Medicine*.

BAKER, P., KAY, A. & WALLS, H. 2014. Trade and investment liberalization and Asia's noncommunicable disease epidemic: a synthesis of data and existing literature. *Globalization and Health,* 10.

BALDWIN, R. 2011. 21st Century regionalism: Filling the gap between 21st Century and 20th Century trade rules. *In:* INSTITUTE, G. (ed.) *Staff Working paper ERSD, World Trade Organisation.*

CITIZEN, P. 2015. Only one of 44 attempts to Use the GATT Article XX/GATS Article XIV ‘General Exception’ Has Ever Succeeded: Replicating the WTO Exception Construct Will Not Provide for an Effective TPP General Exception. Public C. Washington DC: Public Citizen.

COLLIN, J. 2012. Tobacco control, global health policy and development: towards policy coherence in global governance *Tobacco Control,* 21**,** 274-280.

EU. 2016. *CETA chapter by chapter* [Online]. Available: <http://ec.europa.eu/trade/policy/in-focus/ceta/ceta-chapter-by-chapter/> [Accessed December 15 2016].

FOOKS, G. & GILMORE, A. B. 2014. International trade law, plain packaging and tobacco industry political activity: the Trans-Pacific Partnership. *Tobacco Control,* 23**,** e1.

FRIEL, S., GLEESON, D., THOW, A. M., LABONTE, R., STUCKLER, D., KAY, A. & SNOWDON, W. 2013. A new generation of trade policy: potential risks to diet-related health from the trans pacific partnership agreement. *Global Health,* 9**,** 46.

FRIEL, S., HATTERSLEY, L. & TOWNSEND, R. 2015. Trade policy and public health. *Annu Rev Public Health,* 36**,** 325-44.

FRIEL, S., SCHRAM, A. & TOWNSEND, B. 2019. The nexus between international trade, food systems, malnutrition and climate change. *Nature food,* 1**,** 51-58.

GLEESON, D. & FRIEL, S. 2013. Emerging threats to public health from regional trade agreements. *Lancet,* 381**,** 1507-9.

GRUSZCZYNSKI, L. The TBT Agreement and Tobacco Control Regulations. *AJWH,* 8.

GRUSZCZYNSKI, L. 2017. COPing with the global tobacco epidemic: FCTC COP7 and its implications. *European Journal of Risk Regulation,* 8**,** 428-436.

HAWKINS, B. & HOLDEN, C. 2016. A corporate veto on health policy? Global constitutionalism and Investor-State Dispute Settlement. *J Health Polit Policy Law,* 41**,** 969-95.

HIRONO, K., HAIGH, F., GLEESON, D., HARRIS, P. & THOW, A.-M. 2015. Negotiating Healthy Trade in Australia: Health Impact Assessment of the

Proposed Trans-Pacific Partnership Agreement Liverpool, NSW: Centre for Health Equity Training Research and Evaluation, part of the Centre for Primary Health Care and Equity, Faculty of Medicine, UNSW Australia.

KELSEY, J. REGULATORY CHILL: LEARNINGS FROM NEW ZEALAND’S PLAIN PACKAGING TOBACCO LAW *QUT Law Review,* 17**,** 21-45.

KELSEY, J. 2012. The implications of new generation free trade agreements. *Paper to the Global Alcohol Policy Conference.* Bankok.

KELSEY, J. 2013. The Trans-Pacific Partnership Agreement: A Gold-Plated Gift to the Global Tobacco Industry? . *American Journal of Law & Medicine,* 39**,** 237-264.

KHAN, U., PALLOT, R., TAYLOR, D. & KANAVOS, P. 2015. The Transatlantic Trade and Investment Partnership: international trade law, health systems and public health. *The Transatlantic Trade and Investment Partnership: international trade law, health systems and public health***,** 66-pp.

LABONTÉ, R., MOHINDRA, K. S. & LENCUCHA, R. 2011. Framing international trade and chronic disease. *Globalization and Health,* 7.

LABONTÉ, R., RUCKERT, A. & SCHRAM, A. 2017

. Trade, investment and the global economy: Are we entering a new era for health? . *Global Social Policy,* 18**,** 28-44.

LABONTE, R., SCHRAM, A. & RUCKERT, A. 2016. The Trans-Pacific Partnership: Is it everything we feared for health? *Int J Health Policy Manag,* 5**,** 487-496.

LESTER, S. 2015. Domestic Tobacco Regulation and International Law: The Interaction of Trade Agreements and the Framework Convention on Tobacco Control. *Journal of World Trade,* 49**,** 19-47.

MACKENZIE, R. & COLLIN, J. 2012. “Trade policy, not morals or health policy”: the US Trade Representative, tobacco companies and market liberalization in Thailand *Glob SOc Policy,* 12**,** 149-172.

MAMUDU, H. M., HAMMOND, R. & GLANTZ, S. A. 2011. International trade versus public health during the FCTC negotiations, 1999–2003. *Tob Control,* 20**,** e3.

MITCHELL, A., VOON, T. & WHITTLE, D. 2014. Public Health and the Trans-Paciﬁc Partnership Agreement *Asian Journal of international Law***,** 1-31.

O’BRIEN, P. & MITCHELL, A. 2018. On the Bottle: Health Information, Alcohol Labelling and the WTO Technical Barriers to Trade Agreement *QUT Law Review* 1-32.

RIMPEEKOOL, W., SEUBSMAN, S., BANWELL, C., KIRK, M., YIENGPRUGSWAN, V. & SLEIGH, A. 2015. Food and nutrition labelling in Thailand: a long march from subsistence producers to international traders 56**,** 59-66.

RUCKERT, A., SCHRAM, A., LABONTÉ, R., FRIEL, S., GLEESON, D. & THOW, A.-M. 2017. Policy coherence, health and the sustainable development goals: a health impact assessment of the Trans-Pacific Partnership *Critical Public Health,* 27**,** 86-96.

SCHRAM, A., RUCKERT, A., VANDUZER, J. A., FRIEL, S., GLEESON, G., THOW, A.-M., STUCKLER, D. & LABONTE, R. 2018. A conceptual framework for investigating the impacts of international trade and investment agreements on noncommunicable disease risk factors

. *Health Policy and Planning***,** 123-136.

SHEKHAR, S. 2016. Regulatory chill: Taking right to regulate for a spin. Working Paper.: Centre for WTO Studies.

SMITH, K. E., FOOKS, G., COLLIN, J., WEISHAAR, H., MANDAL, S. & GILMORE, A. B. ‘‘Working the System’’—British American Tobacco’s Influence on the European Union Treaty and Its Implications for Policy: An Analysis of Internal Tobacco Industry Documents *PLoS Med,* 7**,** e1000202.

STUMBERG, R. 2013. Safeguards for tobacco control: options for the TPPA. *Am J Law Med,* 39**,** 382-441.

SY, D. K. & STUMBERG, R. K. 2014. TPPA and tobacco control: threats to APEC countries *Tobacco Control,* 23**,** 466-470.

THOW, A.-M., JONES, A., HAWKES, C., ALI, I. & LABONTÉ, R. 2017. Nutrition labelling is a trade policy issue: lessons from an analysis of specific trade concerns at the World Trade Organization *Health Promotion International***,** 1-11.

THOW, A.-M., SNOWDON, W., LABONTÉ, R., GLEESON, D., STUCKLER, D. & HATTERSLEY, L. 2015. Will the next generation of preferential trade and investment agreements undermine prevention of noncommunicable diseases? A prospective policy analysis of the Trans Pacific Partnership Agreement. *Health Policy,* 119.

TOWNSEND, B., GLEESON, D. & LOPERT, R. 2016. The Regional Comprehensive Economic Partnership, intellectual property protection, and access to medicines. *Asia Pac J Public Health,* 28**,** 682-693.

VON TIGERSTROM, B. 2013. How do international trade obligations affect policy options for obesity prevention? Lessons from recent developments in trade and tobacco control. *Can J Diabetes,* 37**,** 182-8.

WALLS, H., BAKER, P. & SMITH, R. 2015. Commentary: Moving towards policy coherence in trade and health. *J Public Health Policy,* 36**,** 491-501.

WALLS, H. & SMITH, R. 2015. Rethinking governance for trade and health. *BMJ,* 351**,** h3652.

WEISS, M. 2015. Trading Health? UK Faculty of Public Health Policy Report on the Transatlantic Trade and Investment Partnership London: UK Faculty of Public Health.

WTO 2013. Briefing note: Regional trade agreements. *9th World Trade Organisation Minsterial Conference.* Bali.

ZEIGLER, D. W. 2009. The alcohol industry and trade agreements: a preliminary assessment. *Addiction,* 104.
